# Dual role of BdMUTE during stomatal development in the model grass *Brachypodium distachyon*

**DOI:** 10.1242/dev.203011

**Published:** 2024-09-26

**Authors:** Roxane P. Spiegelhalder, Lea S. Berg, Tiago D. G. Nunes, Melanie Dörr, Barbara Jesenofsky, Heike Lindner, Michael T. Raissig

**Affiliations:** ^1^ Institute of Plant Sciences (IPS), University of Bern, Altenbergrain 21, 3013 Bern, Switzerland; ^2^ Centre for Organismal Studies (COS), Heidelberg University, Im Neuenheimer Feld 230, 69120 Heidelberg, Germany; ^3^ Oeschger Centre for Climate Change Research (OCCR), University of Bern, Hochschulstrasse 4, 3012 Bern, Switzerland

**Keywords:** BdMUTE, *Brachypodium distachyon*, Grasses, Guard cells, Stomatal development, Subsidiary cells

## Abstract

Grasses form morphologically derived, four-celled stomata, where two dumbbell-shaped guard cells (GCs) are flanked by two lateral subsidiary cells (SCs). This innovative form enables rapid opening and closing kinetics and efficient plant–atmosphere gas exchange. The mobile bHLH transcription factor MUTE is required for SC formation in grasses. Yet whether and how MUTE also regulates GC development and whether MUTE mobility is required for SC recruitment is unclear. Here, we transgenically impaired BdMUTE mobility from GC to SC precursors in the emerging model grass *Brachypodium distachyon*. Our data indicate that reduced BdMUTE mobility severely affected the spatiotemporal coordination of GC and SC development. Furthermore, although BdMUTE has a cell-autonomous role in GC division orientation, complete dumbbell morphogenesis of GCs required SC recruitment. Finally, leaf-level gas exchange measurements showed that dosage-dependent complementation of the four-celled grass morphology was mirrored in a gradual physiological complementation of stomatal kinetics. Together, our work revealed a dual role of grass MUTE in regulating GC division orientation and SC recruitment, which in turn is required for GC morphogenesis and the rapid kinetics of grass stomata.

## Teaser

**Summary:** BdMUTE has a dual role in non-cell-autonomously recruiting subsidiary cells and cell-autonomously orienting guard cell division orientation in *Brachypodium distachyon*.

## INTRODUCTION

Stomata are microscopic pores at the leaf surface that enable plants to control CO_2_ uptake and water vapor loss in leaves sealed with a waxy cuticle (Berry et al., 2010; Chang et al., 2023; Clark et al., 2022). Although cellular arrangements of stomata are diverse among the plant kingdom (Gray et al., 2020; Nguyen and Blatt, 2024; Nunes et al., 2020; Rudall et al., 2013), guard cell (GC) morphology in most phylogenetic clades has not changed from its ancestral, kidney-shaped form (Edwards et al., 1998). Grass GCs, however, show a unique dumbbell shape with elongated, narrow middle parts (i.e. the central rod) and large bulbous apices (Galatis and Apostolakos, 2004; Spiegelhalder and Raissig, 2021; Stebbins and Shah, 1960). Grass stomata also possess lateral, paracytic (i.e. parallel) subsidiary cells (SCs) which are of perigenous origin, meaning they stem from a precursor cell distinct from that of the GCs (Gray et al., 2020; Nguyen and Blatt, 2024; Nunes et al., 2020; Rudall et al., 2017; Stebbins and Shah, 1960). The dumbbell morphology and the presence of SCs were suggested to contribute to faster stomatal movements and improved water-use efficiency (Durney et al., 2023; Franks and Farquhar, 2007; Nunes et al., 2020; Raissig et al., 2017; Raschke and Fellows, 1971).

**Fig. 1.**
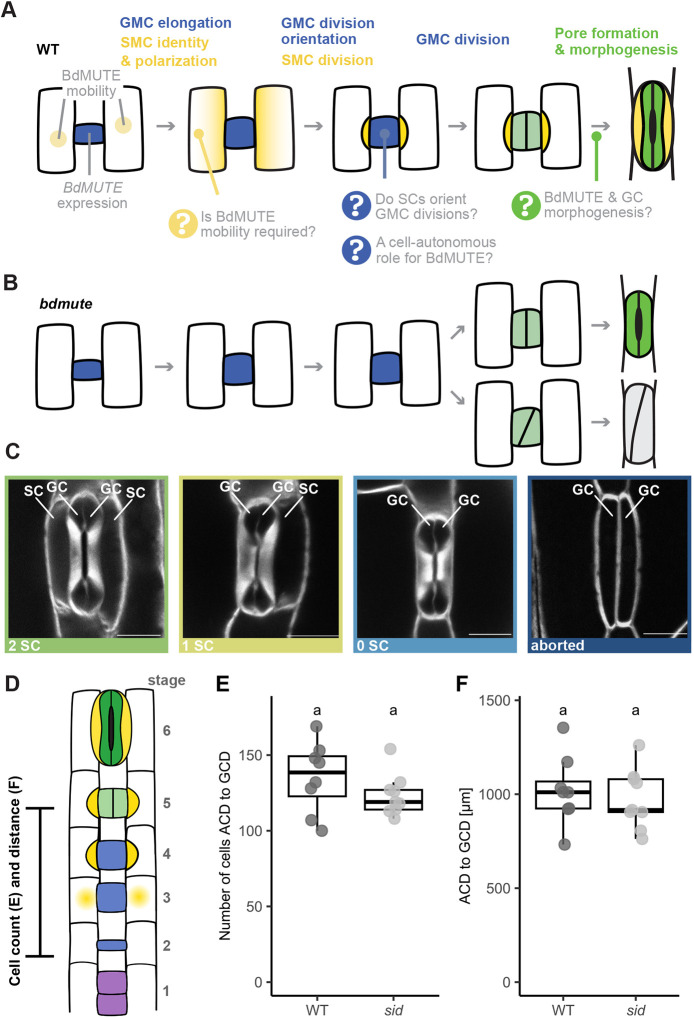
**Potential multiple roles for BdMUTE in subsidiary cell and guard cell formation in the grass stomatal lineage.** (A) Schematic displaying stomatal development in wild type (WT). Open questions regarding the roles and mechanisms of BdMUTE are indicated. (B) In *bdmute* mutants, no subsidiary mother cell (SMC) is established, and no subsidiary cell (SC) division occurs. Guard mother cells (GMCs) mostly divide normally and the guard cell (GC) pair forms a pore and matures into functional complexes (upper GCs, dark green). However, ∼25% of the GMCs show aborted complexes with oblique divisions (lower GCs, gray). (C) Different stomatal morphologies found in WT (2 SCs), and in *sid* (1 SC, 0 SC and arrested phenotype). The images depicting the 1 SC and 0 SC phenotype stem from the weak rescue line M3GM− (see below). Midplane confocal images of fixed, cleared and Direct Red 23-stained mature stomata. Scale bars: 10 μm. (D) Schematic representation of the zone measured in E and F; from first asymmetric cell division (blue, stage 2) to symmetric GC division (light green, stage 5). Developmental stages indicated are: (1) stomatal file initiation, purple; (2) asymmetric division and GMC formation, blue; (3) SMC establishment, yellow gradient; (4) SMC division, yellow; (5) GMC division, light green; (6) GC maturation, dark green. (E,F) Number of cells (E) and distance (F) between the first asymmetric cell division (ACD, stage 2) and the first GMC division (GCD, stage 5) in WT and *sid*. *n*=5-7 individuals per genotype, 1 or 2 stomatal rows per individual (dots are stomatal rows). Box plots show median values (middle bars) and first to third interquartile range (boxes); whiskers indicate 1.5× the interquartile ranges. Significant differences are indicated with lowercase letters to label means, such that bars bearing different letters are statistically different from one another with a minimum *P*-value of <0.05 (two-sided Student's *t*-test).

In grasses, epidermal development is organized in linear cell files, some of which acquire the capacity for stomata formation (Nunes et al., 2020; Raissig et al., 2016; Stebbins and Shah, 1960). Grass stomatal development starts with a transverse asymmetric division specifying the guard mother cell (GMC) which elongates and induces subsidiary mother cell (SMC) fate in the flanking cell files (Fig. 1A). SMCs then undergo an asymmetric longitudinal division to form SCs (Fig. 1A). Then, GMCs divide once symmetrically and longitudinally to form the GCs before complex morphogenetic processes form the pore and generate the dumbbell morphology (Fig. 1A) (Spiegelhalder and Raissig, 2021). Much like in the plant model *Arabidopsis thaliana* (Lee and Bergmann, 2019; McKown and Bergmann, 2020), stomatal development is guided by the conserved set of key basic Helix-Loop-Helix (bHLH) transcription factors SPEECHLESS (SPCH) (Raissig et al., 2016), MUTE (Raissig et al., 2017; Wang et al., 2019; Wu et al., 2019) and FAMA (Liu et al., 2009; McKown et al., 2023) and their heterodimerization partners (Raissig et al., 2016; Wu et al., 2019).

The bHLH transcription factor MUTE holds a key role in guiding grass stomatal development (Nunes et al., 2020; Serna, 2020). Whereas in *Arabidopsis* MUTE determines hydathode pore differentiation, the GMC identity, and is involved in driving the symmetric GMC division (Han et al., 2018; Pillitteri et al., 2007, 2008), the primary role of MUTE in grasses is establishing the SC lineage and driving its longitudinal asymmetric division (Raissig et al., 2017; Wang et al., 2019). Grass MUTE is expressed in GMCs only, but the protein moves laterally into the neighboring cells, establishing SC lineage identity in non-stomatal cell files (Raissig et al., 2017; Wang et al., 2019). In the wild model grass *Brachypodium distachyon* (Raissig and Woods, 2022), most *bdmute* stomata form functional GC complexes that lack SCs (Raissig et al., 2017). In domesticated grasses, however, not only SC but also GC development completely aborts in *mute* mutants as all of the GMCs fail to divide in the proper orientation and no functional stomata are formed (Wang et al., 2019; Wu et al., 2019). This might be an example for domestication-induced loss of genetic diversity causing a lack of developmental compensation. However, much like in domesticated grasses, ∼25% of *bdmute* GMCs in *B. distachyon* show oblique division planes which lead to stomatal abortion (Raissig et al., 2017). And finally, even though functional two-celled complexes form in *bdmute*, the GCs are shorter and develop less of the dumbbell-shaped morphology compared with wild-type stomata flanked by SCs.

This raises open questions regarding the role and functionality of grass MUTE (Fig. 1A). First, is there a cell-autonomous role for MUTE in guiding GC development as suggested by the partial or full GC arrest in wild and domesticated grasses? Is this role primarily pre-mitotic by guiding division plane orientation or also post-mitotic by driving GC differentiation? Or are the observed division plane orientation defects a mere consequence of the lack of SC recruitment? Second, is MUTE mobility indeed required for MUTE function? Or are downstream mobile factors or cell-to-cell signaling responsible for SC formation? Third, is the impaired morphogenesis of *bdmute* GCs a consequence of missing SCs or a missing cell-autonomous role of BdMUTE in the GC lineage?

To address these open questions, we rescued the *subsidiary cell identity defective* (*sid*) mutant (also known as *bdmute-1*) with a 3xGFP-BdMUTE construct with impaired mobility. Scoring the ability of the 3xGFP-BdMUTE to rescue the GC and SC defects, we were able to show that BdMUTE acts cell-autonomously in guiding the GMC division independent of its role in SC formation. GC morphogenesis, however, required the presence of SCs, showing that both cell autonomous and non-cell autonomous aspects influence GC development in *B. distachyon*. Furthermore, our data suggested that physical presence of MUTE in SMCs (i.e. MUTE mobility) is relevant for SC recruitment. 3xGFP tags merely reduced but did not abolish BdMUTE mobility. Therefore, the strongest 3xGFP-BdMUTE line was able to fully rescue both GC and SC defects in mature leaves but caused a severe developmental delay of SC recruitment and spatiotemporal disconnect between GC and SC divisions. Together, our data suggest a dual role of BdMUTE to spatiotemporally synchronize the development of the two stomatal cell types to build physiologically superior, four-celled stomatal complexes in grasses.

## RESULTS

### 3xGFP-BdMUTE rescues *sid* in a dose-dependent manner

**Fig. 2.**
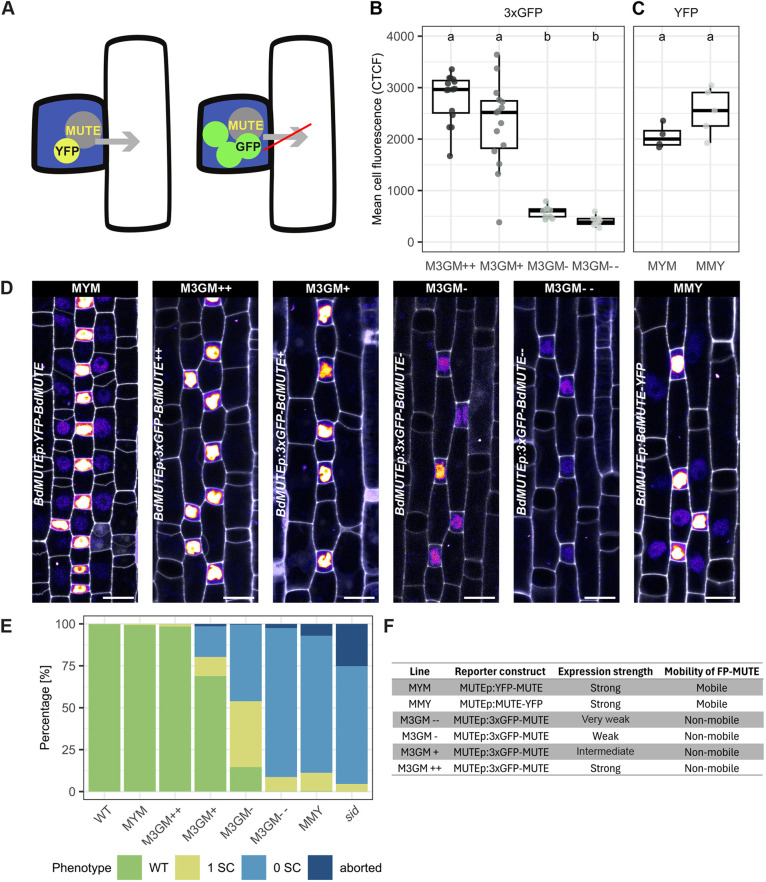
**Expression of mobility impaired 3xGFP-BdMUTE rescues *sid* in a dose-dependent manner.** (A) Schematic displaying the reporter constructs. Single tagged BdMUTE (*sid*;*BdMUTEp:YFP-BdMUTE*, MYM; yellow+gray) can move from GMCs (blue) into lateral cell files (white). A triple GFP tag (*sid;BdMUTEp:3xGFP-BdMUTE*, M3GM; green+gray) impairs BdMUTE mobility and should restrain BdMUTE to GMCs (blue). (B) GFP fluorescence measured in M3GM reporter lines in the *sid* background. Corrected total cell fluorescence (CTCF) of the average of three slices of a *z*-stack around the midplane of the GMC was measured; all images taken with constant laser settings. *n*=8-15 individuals per genotype and 49-105 stomata per genotype (dots are individuals). (C) YFP fluorescence of MYM and sid;*BdMUTEp:BdMUTE-YFP* (MMY). CTCF of the average of three slices of a *z*-stack around the midplane of the GMC was measured; all images taken with constant laser settings. *n*=4-5 individuals per genotype and 29-31 stomata per genotype (dots are individuals). Box plots show median values (middle bars) and first to third interquartile range (boxes); whiskers indicate 1.5× the interquartile ranges. Significant differences are indicated with lowercase letters to label means, such that bars bearing different letters are statistically different from one another (one-way ANOVA followed by Tukey's HSD test; alpha=0.05). (D) Representative midplane confocal images of stage 3 leaf zones of the lines shown in B and C with signal intensity of reporter proteins shown as heatmap and propidium iodide (PI)-stained cell walls shown in gray. MUTE in GMCs shows different intensities in the four GFP lines, mobile signal in the lateral cell files is only visible in the single-tag YFP lines. GFP was imaged with 15% laser and 150% gain, MYM with 12% laser and 100% gain, MMY with 10% laser and 150% gain. Scale bars: 10 µm. (E) Stomatal phenotypes in the mature third leaf of WT, *sid* and *sid* complemented with the different reporter lines shown in D. ‘Aborted’ describes complexes with oblique GMC division or aborted development. *n*=5-12 individuals per genotype and 864-1450 stomata per genotype. Same data shown as boxplots in Fig. S2. (F) Reporter lines used in this study.

MUTE reporters showed very strong signal in GMCs and young GCs (i.e. where it is expressed), and much weaker signal in SMCs and young SCs (i.e. where it moves to) (Raissig et al., 2017; Wang et al., 2019). In *sid* mutants, no SCs are recruited and 25.2%±4.4% (mean±s.d.) of GMCs fail to divide properly and abort (Figs 1B,C, 2E; Fig. S2A). Both phenotypes are fully rescued by a single mCitrine-tagged BdMUTE under its endogenous promoter (*BdMUTEp:mCitrine-BdMUTE* in *sid*=MYM; Fig. 2E,F; Fig. S2A) (Raissig et al., 2017). Yet, whether SC recruitment is needed to time and/or orientate the GMC division remained to be determined (Fig. 1A).

To test whether SC recruitment is required to timely and accurately induce GMC division, we quantitatively analyzed the developmental leaf zone of *sid*. In both wild type (Bd21-3=WT) and *sid*, the number of cells and distance between stage 2 (transverse divisions establishing GMCs) and stage 5 (after GMC division) are equal (Fig. 1D-F). This suggested that SC recruitment is not required to time GMC division and indicated at least partial independence between SMC and GMC divisions. However, whether BdMUTE presence is indeed required in lateral cell files for SC recruitment and whether SC recruitment or a GC-autonomous role of BdMUTE orients the longitudinal, symmetric GMC divisions remained unknown.

To tackle these questions, we generated a mobility-impaired but functional BdMUTE construct (*BdMUTEp:3xGFP-BdMUTE*=M3GM; Fig. 2A,F) and assessed the rescue of GC and SC lineage defects in *sid*. M3GM is supposedly immobile and restricted to the GC lineage, which should allow observing GMC-autonomous roles of BdMUTE. The use of bulky fluorescent protein tags [i.e. adding three (3xFP) instead of just one (1xFP) fluorescent protein] is an established system to prevent protein mobility (Andersen et al., 2018; Clark et al., 2016; Goretti et al., 2020). To test the functionality of 3xGFP-BdMUTE, we first used an overexpression approach (WT; *ZmUBIp:3xGFP-MUTE*) and observed excessive SC-like divisions (Fig. S1) similar to those in single fluorophore overexpression lines (Raissig et al., 2017). We then isolated four independent M3GM lines in the *sid* background and observed severe expression variability (Fig. 2B,D,F). We named the lines according to their expression levels: M3GM−− for the weakest, M3GM− for the weak expressing line, M3GM+ for the strong expressing line, and M3GM++ for the strongest (Fig. 2B,D,F). In addition to these lines, we used WT, *sid*, and *sid* transformed with the mobile, yet non-functional, C-terminally tagged MUTE (*sid*;*BdMUTEp:BdMUTE-mCitrine*=MMY) and the mobile, fully functional, N-terminally tagged rescue construct (MYM; Raissig et al., 2017) as controls (Fig. 2C-F). We quantified corrected total cell fluorescence (CTCF) to determine the expression strength of these lines using the same laser intensities for the GFP lines and a comparable laser setting for YFP lines (see Materials and Methods). MYM and MMY lines show intensities in the same range as the high expressing M3GM++ and + lines (Fig. 2B,C). The weaker expressing lines M3GM− and M3GM− −, however, showed significantly lower intensities (Fig. 2B,D,F).

We quantified the capacity of the six different complementation lines (MYM, MMY and four M3GM) to rescue the GC abortion and SC recruitment phenotypes of *sid* (Fig. 2E; Fig. S2A). We used four different phenotypic classes: if the GCs showed an oblique division and/or are aborted in their development they were classified as ‘aborted’. Longitudinally divided, non-aborted GCs that formed a pore were classified according to the number of SCs they recruited (i.e. zero, one or two SCs; Figs 1C, 2E; Fig. S2A). As shown previously, MYM fully complemented both the GC and SC phenotypes, whereas the mobile, yet non-functional MMY construct failed to complement SC recruitment (Fig. 2E; Fig. S2A). In the third juvenile leaf, though, MMY appeared to partially complement the GC abortion phenotype (Fig. 2E; Fig. S2A), but this was not confirmed in adult leaves (Fig. S7A,B). We, therefore, continued to assume MMY to be non-functional both in terms of SC recruitment and GMC division.

Unexpectedly, we found a dosage-dependent, gradual complementation of both the GC and SC phenotype using the putatively immobile 3xGFP complementation lines (Fig. 2E; Fig. S2A). The weakest line (M3GM−−) complemented the GC abortion phenotype almost completely (25.2%±4.4% aborted GCs in *sid*, 2.5%±2.2% aborted GCs in M3GM−−) but failed to induce additional SCs (Fig. 2E; Fig. S2A). The lines with higher expression (M3GM−, M3GM+) showed a gradual increase in their potential to rescue SC recruitment, with over half of the complexes recruiting at least one SC in the M3GM− line and 68.9% ±38.8% of the complexes recruiting two SCs in the M3GM+ lines (Fig. 2E; Fig. S2A). Strikingly, the strongest line (M3GM++) almost fully rescued the *sid* phenotype, with the mature leaf displaying 98.5%±0.8% WT-like stomata with two SCs, which was statistically indistinguishable from the MYM full complementation lines (99.4%±0.7% two-SC stomata) (Fig. 2E; Fig. S2A). Importantly, this dosage-dependent rescue was confirmed in adult leaves used for gas exchange analysis (see below and Fig. S7A,B). In conclusion, 3xGFP-BdMUTE rescued the GC and SC phenotypes of *sid* in a dose-dependent manner.

### Reduced mobility of 3xGFP-BdMUTE leads to delayed subsidiary cell recruitment

**Fig. 3.**
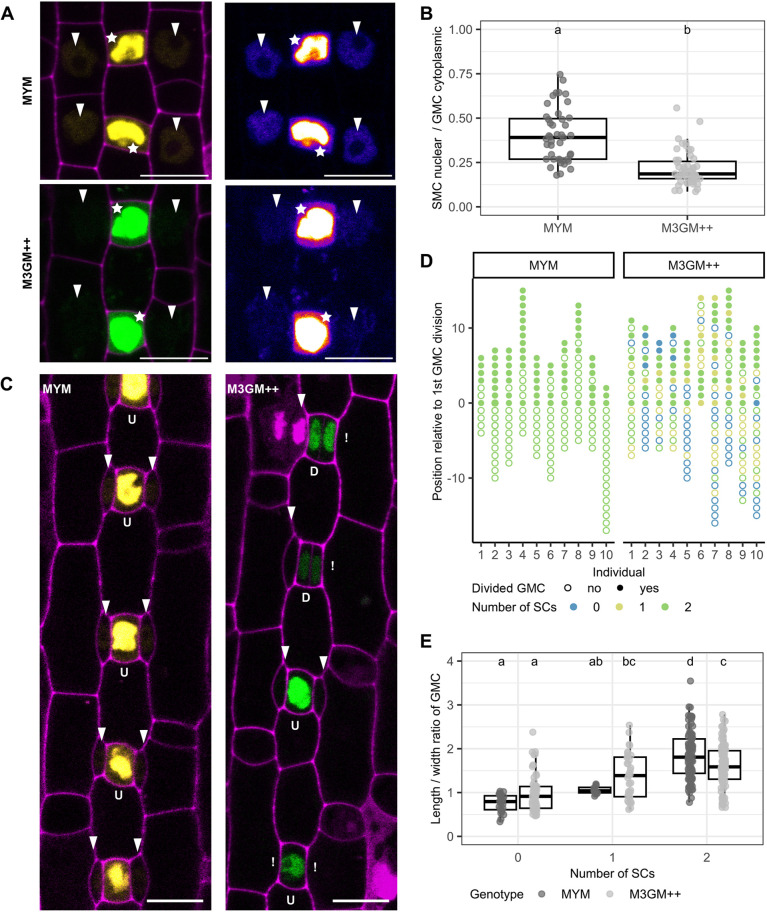
**A triple GFP tag reduces BdMUTE mobility leading to a temporal delay in SC recruitment.** (A) Representative images of guard mother cells (GMCs; stars) and lateral subsidiary mother cells (SMCs; arrowheads) of *sid*;*BdMUTEp:YFP-BdMUTE* (MYM; top panels) and *sid*;*BdMUTEp:3xGFP-BdMUTE* (M3GM++, bottom panels). Cells are imaged with laser intensities that assured non-saturated intensity in cytoplasm of GMCs and visible signal in SMC nuclei. YFP/GFP in yellow/green and propidium iodide (PI)-stained cell walls in purple in the left panels and heatmap projection of YFP/GFP intensity in right panels. (B) Ratio of integrated density of average SMC nuclei signal and average GMC cytoplasmic signal. *n*=2-3 individuals per genotype and 48-58 stomatal complexes per genotype (dots are stomata). (C) Midplane confocal images of second leaf developmental zones in MYM and M3GM++; undivided (U) and divided (D) GMCs and present SCs (arrowhead) or missing SCs (!) are indicated. Cell walls are stained with PI (magenta), fluorophores shown in yellow (YFP) and green (3xGFP). (D) Division and SC recruitment status of GMCs apically and basally relative to the first GMC division in M3GM++ and MYM. Each dot represents one complex and is aligned according to its position relative to the first GMC division. Each column represents a single stomatal row (*n*=10 individuals per genotype). (E) Length/width ratio (LWR) of GMCs in second leaf developmental zones in MYM and M3GM++ flanked by zero, one, or two SCs. *n*=2-6 individuals per genotype and 129-191 stomata per genotype. Box plots show median values (middle bars) and first to third interquartile range (boxes); whiskers indicate 1.5× the interquartile ranges. Significant differences are indicated with lowercase letters to label means, such that bars bearing different letters are statistically different from one another (one-way ANOVA followed by Tukey's HSD test; alpha=0.05). Scale bars: 10 µm.

The dosage-dependent rescue of SC recruitment by mobility-impaired 3xGFP-BdMUTE implied that BdMUTE mobility might not be required to induce SC identity and divisions. However, careful, quantitative confocal imaging of the strongest 3xGFP-BdMUTE line (M3GM++) revealed a weak 3xGFP-BdMUTE signal in SMCs (Fig. 3A). This suggested that MUTE mobility is not completely abolished but merely reduced by a 3xGFP tag. In the M3GM+ line this signal was not detectable anymore (Fig. S2B), yet we cannot fully exclude 3xGFP-BdMUTE signal below the detection limit in all M3GM lines. To quantify BdMUTE mobility, we imaged MYM and M3GM++, which both appeared to fully complement the GC and SC phenotype. Laser intensity was adjusted to visualize signal in the SMC nuclei without achieving saturation in the GMC cytoplasm (Fig. 3A). GMC nuclear signal was saturated under these conditions (Fig. 3A). We then measured the integrated density of signal in a constant area in the GMC cytoplasm and the SMC nuclei and used the ratio of these intensities as a proxy for BdMUTE mobility from the GMC to the SC lineage (Fig. 3B). The SMC nuclear to GMC cytoplasmic signal ratio was twice as high for YFP-BdMUTE (0.403±0.151) than for 3xGFP-BdMUTE (0.216±0.097; Fig. 3B). This suggested that BdMUTE mobility – albeit significantly reduced – was not completely abolished when a 3xGFP tag was added (Fig. 3B). Furthermore, RNA-fluorescence *in situ*-hybridization (RNA-FISH) against the endogenous *BdMUTE* mRNA indicates that it is the BdMUTE protein that is mobile rather than the mRNA (Fig. S3A-C). However, we cannot fully exclude that mRNA levels are below the detection limit in SMCs (see also Discussion).

Even though the mature leaf epidermis of M3GM++ lines suggested a full rescue, we observed a significant delay of SC recruitment in these lines (Fig. 3C,D). In complementing MYM lines, 100% of dividing GMCs have successfully recruited both SCs (Fig. 3C,D). In M3GM++ lines, however, many dividing GMCs recruited only one or no SCs yet (Fig. 3C,D). This suggested that the spatiotemporal order of GC and SC divisions, where the SC divisions always precede the symmetric GMC divisions, is disturbed when BdMUTE mobility is impaired. To quantify the delay in M3GM++ compared with MYM we used two different approaches. First, we analyzed up to 15 developing stomatal complexes apically (i.e. older) and basally (i.e. younger) relative to the first GMC division in different stomatal files of MYM and M3GM++ (Fig. 3D) and scored if these GMCs had already recruited one or two SCs. In all analyzed stomatal files in MYM, 100% of the cells above and below the first GMC division were already flanked by two SCs (Fig. 3D). In M3GM++, only 43% of the cells (*n*=89/205 GMCs) had already recruited two SCs, 26% (*n*=54/205 GMCs) had only recruited one SC and 30% (*n*=62/205 GMCs) did not recruit any SCs (Fig. 3D). The fraction of GMCs that completed the symmetric division apically to the first GMC division, however, was similar between the two lines (*n*=73/94 GMCs=78% in MYM; *n*=81/118 GMCs=69% in M3GM++; Fig. 3D). Second, the length/width ratio (LWR) of GMCs is a good proxy for the specific developmental stage between stage 3 and stage 5 (Fig. 1D) (Zhang et al., 2022). We, therefore, measured the LWR of GMCs and correlated it to the SC recruitment status (zero, one or two SCs; Fig. 3E) and GMC division (yes or no; Fig. S3D). GMC LWR before GMC division was not different between MYM and M3GM++ (Fig. S3D) suggesting that GC development was not disturbed. However, only MYM lines showed SC recruitment to be tightly regulated in time and space (Fig. 3E). GMCs in MYM that only recruited one SC showed a narrow window of GMC LWR (1.06±0.09) and were not very frequent (9%; Fig. 3E). GMCs with a lower LWR (<1.0) recruited no SCs yet, and GMCs with a higher LWR (>1.2) recruited two SCs already (Fig. 3E). In M3GM++, the GMCs that recruited none or only one SC show a wide distribution of LWRs (0.98±0.42 and 1.38±0.52, respectively, in M3GM++ compared with 0.76±0.20 and 1.06±0.09 in MYM; Fig. 3E). This indicated that the correlation between GMC LWR and number of recruited SCs was severely disturbed in M3GM++, suggesting a significant SC recruitment delay when BdMUTE mobility is impaired. However, even reduced BdMUTE mobility appeared to be sufficient to eventually induce SMC identity and trigger SMC division. This might also explain the strict dosage dependency of 3xGFP-BdMUTE in its ability to complement the SC recruitment defects; weakly expressed lines might not reach sufficient BdMUTE levels in neighboring cells to establish SMC identity.

Together, the spatiotemporal disconnect between GC and SC development when BdMUTE mobility is reduced reinforces the concept of BdMUTE mobility being required to establish SMCs and induce SC divisions. Although we could not completely abolish BdMUTE mobility using a bulky 3xGFP tag, the reduction of mobility was sufficient to severely disturb and delay SC recruitment.

### A cell-autonomous role for BdMUTE in guard mother cell division orientation independent of subsidiary cell recruitment

Another implication of the dosage-dependent 3xGFP-BdMUTE complementation is that weak 3xGFP-BdMUTE expression in the GMC was sufficient to rescue GC abortion irrespective of successful SC recruitment. Although GC abortion was almost completely rescued in M3GM−− (Fig. 2E; Fig. S2), this line never formed two SCs and only weakly increased the frequency of single SC complexes from 4.4%±0.5% in *sid* to 8.4%±2.7% in M3GM−−. Consequently, the relevant aspect in rescuing the GMC division phenotype appeared to be BdMUTE presence in the GMC (Fig. 2D,E; Fig. S2). This suggested that proper GMC division and formation of functional GCs did not depend on successful SC recruitment, that GMC and SC divisions are two independent processes, and that BdMUTE has a cell-autonomous role during GC formation.

**Fig. 4.**
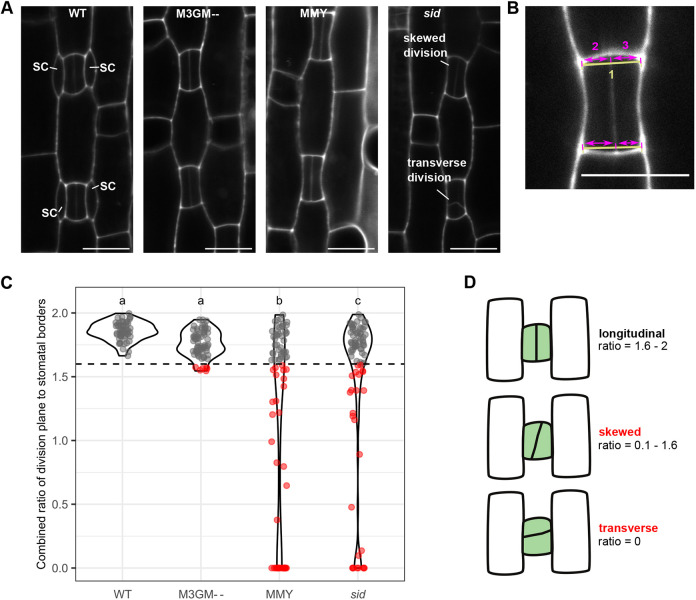
**Low BdMUTE levels in guard mother cells mostly rescue the GMC division plane orientation defects.** (A) Representative images of recently divided guard mother cells (GMCs) in wild type (WT), *sid*;*BdMUTEp:3xGFP-BdMUTE* (M3GM−−), *sid*;*BdMUTEp:BdMUTE-YFP* (MMY) and *sid*. Midplane confocal images of developmental zones stained with propidium iodide (PI). (B) Schematic of division plane orientation measurements. A line was drawn to connect the corners of the GMC at the apical and basal side (1). The distance from each corner to the intersection with the GMC division plane was measured along the line (2 and 3). The smaller distance divided by the longer distance resulted in the ‘skewness’ ratio of the apical (Fig. S4A,B) and basal side (Fig. S4C,D). Scale bars: 10 μm. (C) Total ‘skewness’ ratios per GMC as a sum of apical and basal ‘skewness’ ratios. A total ratio <1.6 (dashed horizontal line) represents a skewed division (indicated as red dots). *n*=7-8 individuals per genotype and 66-82 stomata per genotype (dots are stomata). Significant differences are indicated with lowercase letters to label means, such that bars bearing different letters are statistically different from one another (one-way ANOVA followed by Tukey's HSD test; alpha=0.05). (D) Schematic representation of the division phenotypes depicted in C. Longitudinal division represented by ratios >1.6 (i.e. all WT stomata) were used as a WT cutoff. GCs with a division plane orientation <1.6 and >0 were classified as ‘skewed’ and a transverse division is shown as ratio=0.

Many of the aborted complexes in *sid* showed skewed or transverse divisions (Fig. 4A). Therefore, BdMUTE might have a function in orienting GMC divisions, and wrongly specified division planes and a non-symmetric division might cause GC abortion. We thus quantified GMC division orientation in WT, *sid*, MMY, and the weakest, non-mobile, M3GM−− line (Fig. 4; Fig. S4). We imaged developmental zones of these lines right after the GMC division (early stage 5; Fig. 1D) and measured the distances of the central wall to lateral GC walls (Fig. 4B) on the apical and basal side of the complex (Fig. S4). We then calculated the ratio of the shorter to longer distance in all four lines (Fig. 4B; Fig. S4). A ratio of 1 would indicate a perfectly symmetrical division (in the following termed ‘longitudinal’), a ratio smaller than 1 indicates a skewed division. Completely transverse divisions were classified as a ratio of 0 (Fig. S4). We assumed that one side exhibiting skewed division may be sufficient to cause GC complex abortion, therefore we added the top and bottom ratios to investigate the ‘total skewness’ per complex (Fig. 4C,D). Accordingly, a ratio of 2 would represent perfectly symmetrical divisions. Stage 5 GC complexes in WT all showed a ‘total skewness’ ratio range of 1.6-2 (Fig. 4C). As no stage 5 GC complexes in WT abort, we defined this range as sufficiently symmetric to form functional GC complexes (Fig. 4C, gray dots). Many stage 5 complexes showed lower ‘total skewness’ ratios in *sid* (26/82 complexes below 1.6; 31.7%) and *sid* transformed with MMY (37/78; 47.4%). In M3GM−−; however, the ‘total skewness’ ratio was almost fully rescued, with only 9.1% (6/66) of stage 5 GC complexes showing a ratio smaller than 1.6, and none smaller than 1.5 (Fig. 4C).

In conclusion, our data suggested that weak expression of 3xGFP-BdMUTE in GC is sufficient to cell-autonomously rescue division plane orientation without requiring SC recruitment. Furthermore, division plane orientation defects appear to be strongly correlated with GC complex abortion in *sid* and thus potentially causative for failed GC development in grass *mute* mutants. However, more GMCs display a skewness ratio smaller than 1.6 than actually abort, suggesting that slightly skewed GMC divisions can nonetheless result in functional GC complexes.

### Dumbbell guard cell morphogenesis requires subsidiary cell recruitment

**Fig. 5.**
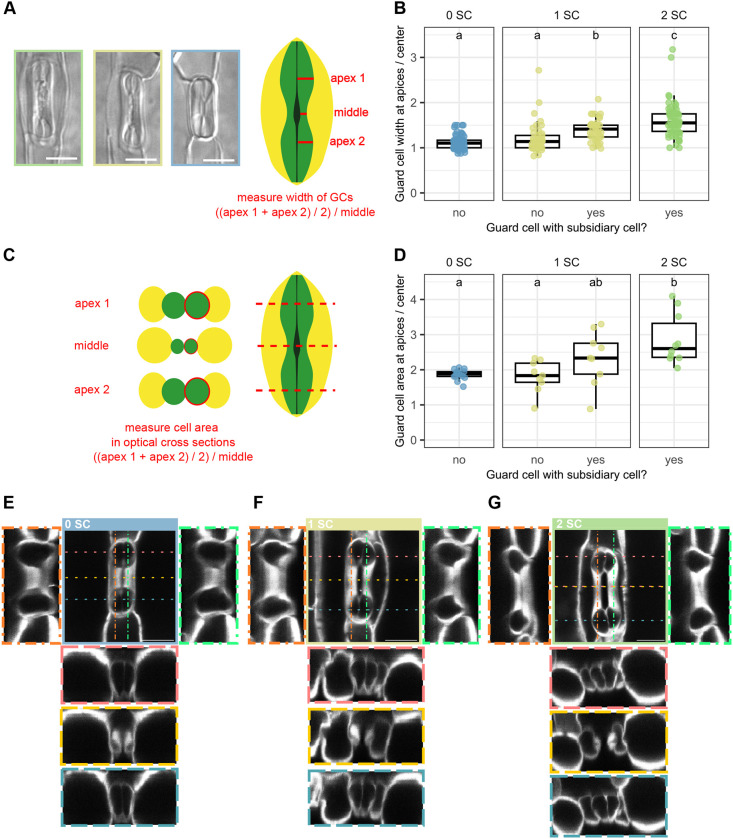
**Presence of subsidiary cells influences guard cell morphology.** (A) Differential interference contrast (DIC) images of stomatal morphologies of the sid;BdMUTEp:3xGFP-BdMUTE (M3GM−) line, showing complexes with two, one or no subsidiary cells (SCs) (green, yellow and blue box, respectively). The schematic indicates how these complexes were measured to calculate a curvature ratio in B. (B) Guard cell (GC) width at the apex/middle ratio of M3GM− GCs flanked by either zero, one or two SCs. Ratio indicates curvature of GCs; each data point represents one cell, measurements performed on DIC images as shown in A. *n*=2 individuals and 16-36 stomata per phenotype. (C) Schematic illustrating analysis of optical cross-sections (shown in E-G) to calculate curvature ratio of cross-section areas in D. (D) Quantification of average apex/middle area ratio of GCs with zero, one or two SCs in M3GM−. *xz* cross-sections were measured as shown in C (see E-G). Each dot represents one GC. *n*=21 complexes. (E-G) 3D confocal stacks of mature stomatal complexes of the M3GM− line without SCs (E), with one SC (F) and with two SCs (G). *xz* cross-sections through the apices and middle and *yz* sections are displayed and indicated by dashed, colored lines and respective frames. Confocal images are of fixed, cleared and Direct Red 23-stained stomata. Box plots show median values (middle bars) and first to third interquartile range (boxes); whiskers indicate 1.5× the interquartile ranges. Significant differences are indicated with lowercase letters to label means, such that bars bearing different letters are statistically different from one another (one-way ANOVA followed by Tukey's HSD test; alpha=0.05). Scale bars: 10 μm.

So far, our data suggested that the division programs of the GC and SC lineage are largely independent of one another, with BdMUTE controlling two independent aspects in the respective lineages. Yet, GCs that are not flanked by SCs displayed distinct morphologies and are stockier and rounder with a less pronounced dumbbell shape (Fig. 5; Zhang et al., 2022; Durney et al., 2023; Liu et al., 2024). GCs flanked by SCs, on the other hand, displayed the characteristic dumbbell shape of grass GCs (Fig. 5). To quantify this, we used mature leaves of the M3GM− line that showed the largest diversity of SC recruitment phenotypes, with almost equal parts recruiting zero, one or two SCs (Fig. 2E; Fig. S2A, Fig. S7A,B). We quantified total GC length and width at apices and in the central rods of stomatal complexes with zero, one and two SCs (Fig. 5A,B; Fig. S5). Stomatal complexes with two SCs were significantly longer than those without or with only one SC, which showed intermediate GC lengths (Fig. S5). We calculated the apex/middle ratio (AMR) as a proxy for curvature of the GCs and found a significant difference between GCs with and without SCs (Fig. 5A,B). In a stomatal complex without SCs, the GCs had a lower mean AMR (1.10±0.13) than GCs in a stomatal complex with two SCs (1.56±0.24; Fig. 5B). This suggested that morphogenesis either depended on the presence of a flanking SC or different levels of functional BdMUTE protein in GMCs and a cell-autonomous role of BdMUTE in GC morphogenesis. Yet, even within a single complex that only recruited one SC, the morphology of the two GCs was starkly different; the GC flanked by an SC showed a higher AMR (1.30±0.19) compared with the non-flanked GC (1.11±0.20; Fig. 5B). In these cases, both GCs stem from the same GMC, and hence likely inherited the same amount of BdMUTE protein. Therefore, investigating GC shape in such complexes allowed us to uncouple GC morphogenesis from the presence of BdMUTE as both GCs had similar amounts of BdMUTE but only the presence of a neighboring SC lead to a more elongated dumbbell shape. This strongly suggested a non-cell-autonomous effect of the SCs on GC morphogenesis independent of BdMUTE. However, a single SC was not sufficient to fully reconstitute the AMR of the flanked GC (Fig. 5B) nor the GC length (Fig. S5). The GC length of complexes with a single SC (21.4 µm±1.64 µm) was between that of complexes with two SCs (24.7 µm±2.59 µm) or no SCs (19.6 µm±2.01 µm; Fig. S5). This indicates that GC elongation and morphogenesis both depend on recruitment of two lateral SCs and that they might be functionally linked.

An issue of using brightfield DIC images to quantify AMRs of GCs is that the focal plane of individual stoma is difficult to control, potentially introducing a measurement bias. Therefore, we generated 3D confocal stacks of individual stomata with no, one or two SCs in M3GM− (Fig. 5E-G). We then quantified the GC area of optical *xz* cross-sections of the central rod and the two apices of M3GM− complexes with zero, one and two SCs (Fig. 5C,D). GCs that are not flanked by SCs showed a consistent mean GC circumference at the AMR irrespective of whether they were part of a stomatal complex that recruited no or one SC (1.81±0.45 in complexes with one SC and 1.86±0.15 in complexes with zero SCs; Fig. 5D). In contrast, GCs that were flanked by an SC in complexes with only one SC showed a higher AMR (2.33±0.77; Fig. 5D). However, GCs from WT-like (in M3GM−) stomatal complexes with two SCs showed the highest area AMR (2.86±0.72) and thus the most pronounced dumbbell shape (Fig. 5D).

Together, this suggests that dumbbell morphogenesis is indeed non-cell-autonomously regulated by a flanking SC. Yet, when only one SC is recruited, morphogenesis of the flanked GC is incomplete, which might be related to impaired GC elongation in single SC complexes.

### Gradual complementation of the *sid* mutant phenotype is quantitatively reflected in leaf-level gas exchange physiology

**Fig. 6.**
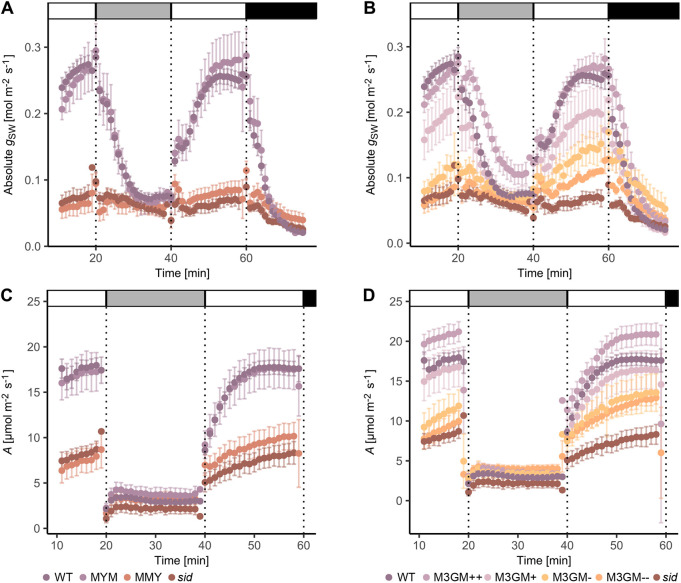
**Physiological complementation follows cytological complementation.** (A) Absolute stomatal conductance (*g*_SW_) of wild type (WT), *sid*;*BdMUTEp:YFP-BdMUTE* (MYM), *sid*;*BdMUTEp:BdMUTE-YFP* (MMY) and *sid.* (B) *g*_SW_ of WT, the four *sid*;*BdMUTEp:3xGFP-BdMUTE* (M3GM) lines (++, +,− and −−) and *sid.* (C) Carbon assimilation (*A*) of WT, MYM, MMY and *sid.* (D) *A* of WT, M3GM lines and *sid.* Note that the same WT and *sid* data is shown in A, C and B, D. Measured were the youngest, fully expanded leaves of 3- to 4-week-old, soil-grown plants; *n*=5-6 individuals per genotype, 26 individuals for WT. Grayscale bars indicate changing light conditions (PPFD: white=1000 µmol m^−2^ s^−1^, light gray=100 µmol m^−2^ s^−1^, black=0 µmol m^−2^ s^−1^). Dots represent the means of all individuals and error bars indicate standard error. Data depicted is used to calculate the steady state values in Table 1 and Fig. S6. The adult leaves assessed for gas exchange were also used to quantify the complementation of *sid*-induced stomatal morphological defects of the different complementation lines and to calculate stomatal densities (Table 1; Fig. S7).

In *sid* plants, absolute stomatal conductance (*g*_sw_), speed of stomatal movements, maximum stomatal aperture and maximum stomatal conductance were drastically impaired (Raissig et al., 2017). To test whether gradual rescue of the GC and SC defects in *sid* was reflected in stomatal gas exchange parameters, leaf-level gas exchange was investigated under fluctuating light intensities. For WT, MYM, MMY, *sid* and the four M3GM (−− to ++) lines, we measured and calculated *g*_sw_ and carbon assimilation (*A*). As reported previously, *sid* (and also the non-functional, non-complementing MYM) shows significantly reduced *g*_sw_ and *A* in high light conditions in comparison with WT (and the functional, complementing MYM) (1000 µmol m^−2^ s^−1^; Fig. 6A,C; Table 1; Fig. S6). Strikingly, the dosage-dependent, gradual complementation of stomatal morphology in the M3GM lines is fully mirrored in their respective *g*_sw_ and *A* (Fig. 6B,D; Table 1; Fig. S6). This confirms the quantitative importance of stomatal morphology and presence of SCs on gas exchange in grasses. Stomatal density, despite being slightly different in some lines, does not explain the major significant differences in *g*_sw_ and *A* as verified by calculating physiological parameters per stoma (Figs S7C, S8)*.* Stomatal morphology of the leaves used for gas exchange measurements, however, quantitatively recapitulated the phenotypes observed in the third leaf (Fig. 2E; Figs S2, S7A,B). Therefore, our data strongly suggested that stomatal morphology rather than density affected gas exchange levels in the assessed lines in a quantitative manner, with WT-like complexes allowing for a higher and wider range of *g*_sw_ and *A*.

**Table 1.**
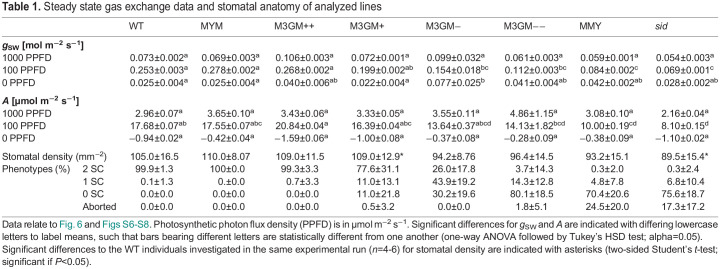
Steady state gas exchange data and stomatal anatomy of analyzed lines

## DISCUSSION

In this study, we addressed open questions regarding the function of BdMUTE during SC and GC formation in grasses (Fig. 1). We were able to show that reducing BdMUTE mobility resulted in spatiotemporally delayed SC recruitment, indicating that BdMUTE mobility is indeed required to recruit SCs (Fig. 3). In addition, BdMUTE guides GC division orientation in a cell-autonomous manner (Fig. 4) that does not require SC recruitment (Fig. 2). However, complete morphogenesis of dumbbell GCs requires bilateral SC recruitment (Fig. 5). Finally, the dose-dependent rescue of GC and SC formation in *sid* by 3xGFP-BdMUTE (Fig. 2) was mirrored in a gradual complementation of stomatal physiology (Fig. 6), quantitatively linking stomatal anatomy with function. The distinct origin of GCs and SCs and the strict developmental gradient of stomatal development in *B. distachyon* allowed us to further untangle the complex, multifaceted roles of BdMUTE during grass stomatal formation.

### Subsidiary cell recruitment is linked to MUTE mobility

Grass MUTE protein was shown to be mobile, being expressed in GMCs and moving laterally into the neighboring cell files (Raissig et al., 2017; Wang et al., 2019). Although lateral deployment of a core stomatal transcription factor through cell-to-cell mobility to induce ‘ectopic’ stomatal fate is a compelling hypothesis, it still lacked experimental proof. We attempted to immobilize BdMUTE by tagging it with 3xGFP to hinder protein mobility (Andersen et al., 2018; Clark et al., 2016; Goretti et al., 2020) but saw full rescue of *sid* in lines with high protein dosage. This initially suggested that SC recruitment is not dependent on BdMUTE presence in the lateral cell files, but rather relying on secondary, putatively mobile factors downstream of MUTE.

However, careful imaging revealed reduced but not abolished mobility that, within a window of opportunity, was sufficient to reach the required threshold to induce SC divisions (Fig. 3C-E). Other studies reported a steric 3xGFP tag as sufficient to immobilize proteins (Andersen et al., 2018; Clark et al., 2016; Goretti et al., 2020; Lucas et al., 1995). This could be due to a more narrow window of opportunity in which reduced mobility is sufficient to prevent the protein from reaching the required threshold and activate a specific developmental process. Alternatively, plasmodesmata in different tissues or species might have distinct size exclusion limits affecting the degree of protein mobility impairment by adding 3xGFP (Bayer and Benitez-Alfonso, 2024). Also, different cell types and tissues might express distinct proteins that promote cell-to-cell mobility like SHORTROOT INTERACTING EMBRYONIC LETHAL (SIEL), which can interact with different mobile transcription factors to promote their mobility (Koizumi et al., 2011). Finally, we cannot fully exclude cleavage of the 3xGFP fusion protein or 3xGFP-MUTE protein presence below our detection limit, both of which could only be tested with a BdMUTE-specific antibody and a highly sensitive assay.

Furthermore, RNA-FISH (Fig. S3A-C) indicated that it is in fact the BdMUTE protein that moves to lateral files and not the mRNA. This makes sense given the fact that mRNAs, despite the common misconception of being rather small, are usually much bigger than their protein counterpart. A single nucleotide is about three times the size of an amino acid: three nucleotides are coding for one amino acid and the mRNA contains untranslated regions and can also form 3D structures (Milo and Phillips, 2015). It is thus estimated that an mRNA has a 10-fold spatial extent compared with the protein it codes for (Milo and Phillips, 2015) making it physically much more likely that proteins rather than mRNAs move through plasmodesmata.

Taken together, our results strongly suggest that BdMUTE must be present in both GMCs and SMCs to spatiotemporally coordinate SC recruitment and GMC division, which are two largely independent processes. Strikingly, we find large temporal flexibility regarding the time point of SC formation. Even severely delayed SC formation in M3GM++ leads to WT stomata in mature leaf zones. However, as GC morphogenesis and complex maturation cannot be fully uncoupled from the presence of SCs, we cannot exclude that delayed SC recruitment can quantitatively affect GC morphogenesis. Even slight disturbances in the GC maturation process could have functional consequences.

### BdMUTE guides guard cell division plane orientation in a cell-autonomous manner

In contrast with the stomatal development in *Arabidopsis*, which relies on self-renewal of meristemoids (Bergmann and Sack, 2007; Cheng and Raissig, 2023; Lau and Bergmann, 2012), stomatal development in grasses is restricted to the most basal part of the developing leaf and takes place in specialized cell files (Stebbins and Shah, 1960). The first asymmetric cell division (ACD) directly gives rise to a GMC, omitting the meristemoid stage (Raissig et al., 2016).

Thus, division capacity in the grass leaf epidermal lineages is fundamentally different and spatially restricted to the leaf base and to stage 1 and stage 2 stomatal cells (Fig. 1D). SMC and GMC divisions both occur beyond the ‘transverse division competency zone’. Therefore, GC fate commitment in grasses might not require division competency inhibition like in eudicots but a division competency establishment instead (McKown and Bergmann, 2020; Nunes et al., 2020). Indeed, FAMA proteins in grasses lack the conserved motif that mediates the interaction with the cell cycle inhibitor RBR1 that is involved in repressing division competency in GCs, and show arrested stomata after the symmetric GMC division but no additional divisions (Matos et al., 2014; Spiegelhalder and Raissig, 2021; Liu et al., 2009; McKown et al., 2023; Wu et al., 2019). We can only speculate what establishes and regulates division competency in the grass GC lineage. Clearly, *mute* mutant GMCs can divide in both wild and domesticated grasses, but fail to define division plane orientation (Raissig et al., 2017; Wang et al., 2019; Wu et al., 2019). Potentially, the duplicated SPCH genes regulate division competency both in stage 1 stomatal cells and in GMCs as they are expressed in these stages (Raissig et al., 2016). We observed no impaired cell division capacity in the GC lineage in *sid*, suggesting that no division defects in stage 1/stage 2 stomatal cells occur in *sid*. In the SC lineage, however, the establishment of SMC identity is inherently linked to division competency. Therefore, MUTE does regulate division competency in SMCs, as SC divisions rarely occur in *mute* mutants (Raissig et al., 2017; Wang et al., 2019; Wu et al., 2019).

Both, the highly asymmetric divisions we observed in SMCs and the symmetric divisions of the GMC are unique in the grass epidermis as they are some of the rare longitudinal divisions. Grass MUTE protein – being present in both GMCs and SMCs – might be linked to the specification of longitudinal division orientation. Indeed, MUTE expression in GMCs, but not SC recruitment, is required for GMC division orientation (Figs 2E, 4; Figs S2A, S7A,B) as has been previously hypothesized, but not experimentally shown (Serna, 2021). In SMCs, MUTE induces BdPOLAR (Zhang et al., 2022), a distal polarity program that together with a MUTE-independent proximal program (Cartwright et al., 2009; Facette et al., 2015; Humphries et al., 2011; Zhang et al., 2012, 2022) guides the ACD of SMCs. In particular, the distal BdPOLAR domain, which is absent in *bdmute* mutants, appears to regulate cortical division site orientation linking BdMUTE to a division orientation program in SMCs (Zhang et al., 2022). Yet, BdPOLAR is normally expressed in *bdmute* GMCs, suggesting that a distinct program orients division planes in GMCs (Zhang et al., 2022). Specific cyclins, for example, were shown to be involved in both symmetric cell division (SCD) (Han et al., 2018; Weimer et al., 2018) and ACD (Cruz-Ramírez et al., 2012) during stomatal development, and MUTE has been shown to regulate expression of such cell-cycle factors (Han et al., 2018; Weimer et al., 2018; Zuch et al., 2023). Therefore, we propose a grass GMC-specific cell division program, in which the SPCHs establish division capacity and MUTE controls division orientation.

### Subsidiary cells influence guard cell morphogenesis

In *sid*, GCs are shorter and stockier with a less pronounced dumbbell shape (Figs 1C, 5; Fig. S5). We observed partial reconstitution of the GC shape (AMR) and length in stomatal complexes with just one SC, yet only those GCs that were actually flanked by the SC reconstituted shape (Fig. 5). However, GC morphogenesis and elongation is only complete when both GCs are flanked by SCs. Indeed, GC shape is also compromised in SMC polarity mutants that fail to recruit SCs at high frequencies (Zhang et al., 2022; Liu et al., 2024). This suggests that the presence of lateral SCs non-cell autonomously influences GC shape independent of MUTE protein levels in GMCs. Whether this involves cell-to-cell signaling processes, biomechanical processes and/or the specific maturation processes of the shared apoplast between GCs and SCs remains to be determined. In mature grass stomata, GCs and SCs are thought to oppositely shuffle osmolytes and thus execute opposite turgor pressures facilitating rapid opening and closing kinetics (Durney et al., 2023; Franks and Farquhar, 2007). Potentially, active pressurization of the SCs during stomatal development might support GC elongation and morphogenesis. Biomechanical processes were shown to influence plant cell morphogenesis (Elliott et al., 2024; Geitmann and Ortega, 2009; Landrein and Hamant, 2013; Rui et al., 2019; Yi et al., 2019), indicating that mechanical influence of the SCs could be at least partially involved in guiding the morphogenesis of grass GCs.

In GCs without an SC, we observed a thicker middle rod with more pronounced cell wall staining, which indicates excessive cell wall formation (Fig. 5E). This could be a byproduct of less elongated GCs and, consequently, a surplus of cell wall material. In stomatal complexes with a single SC, however, both GCs were the same length (Fig. S5), but we still primarily observed a stronger cell wall signal in GCs that were not flanked by SCs. Thus, cell wall build up might indicate a form of compensatory effect, possibly induced by altered biomechanical interactions with flanking pavement cells instead of SCs.

### The dose-dependent cytological rescue is mirrored by a dose-dependent physiological rescue

Different levels of 3xGFP-MUTE caused a gradual, dosage-dependent rescue of both GC and SC division phenotypes that was quantitatively reflected in the gradual rescue of the stomatal physiological defects of *sid* (Fig. 6; Figs S6-8).

Stomatal density was shown to affect leaf-level gas exchange in WT *Brachypodium* (Nunes et al., 2022) and transgenic cereal crops (Caine et al., 2018; Dunn et al., 2019; Hughes et al., 2017; Karavolias et al., 2023). Out of all lines in this study, only *sid* showed a significantly decreased stomatal density in comparison with the simultaneously grown WT plants (Fig. S7C) suggesting that the gradually lower *g*_SW_ and *A* under high light conditions in the M3GM lines was unlikely due to this anatomical trait (Fig. 6; Fig. S6). In addition, the gradual differences remained even when the gas exchange parameters were calculated per stoma (Fig. S8), further indicating a more dominant role of stomatal anatomy rather than density on gas exchange.

However, it remains unclear whether the decreased stomatal performance in the lines described here is due to the lack of SCs or is caused by altered GC morphology and size, as these traits likely regulate the rapid stomatal movements in a synergistic manner (Franks and Farquhar, 2007; McAusland et al., 2016; Nunes et al., 2020).

Together, we were able to show that BdMUTE cell-autonomously guides the orientation of the GMC division in stomatal development and leveraged partial *bdmute* complementation lines to quantitatively investigate the effect of SCs on GC morphology. Combining these findings with physiological measurements across a gradient of complementation further allowed us to link stomatal form with function in a density-independent context.

## MATERIALS AND METHODS

### Plant material and growth conditions

Bd21-3 *B. distachyon* was used as WT. Second leaf confocal microscopy was conducted on plate-germinated seedlings that were sterilized with 20% bleach, 0.1% Triton X-100, washed and placed on ½ MS Murashige & Skoog (Duchefa) plates. After 2 days of vernalization and stratification (in dark, 4°C), these seeds were placed into a 28°C chamber with a 16 h light/8 h dark cycle and 110 μmol m^−2^ s^–1^ (as the unit of photosynthetically active photon flux density, PPFD) for 5-6 days until imaging.

After second leaf microscopy, the seedlings were planted in soil, consisting of four parts ED CL73 (Einheitserde) and one part Vermiculite and grown in a greenhouse or growth chamber with a 18 h light/6 h dark cycle (PPFD 250-350 μmol m^−2^ s^–1^; day temperature 28°C, night temperature 22°C); (also described in Haas and Raissig, 2020; Nunes et al., 2022). The *sid* line described in Raissig et al. (2017) was used as a mutant line and background for reporter constructs.

### Generation of reporter constructs

Reporter constructs were used as described in Raissig et al. (2017) (*BdMUTEp:YFP-BdMUTE*, *BdMUTEp:BdMUTE-YFP*) or generated using the GreenGate cloning system (Lampropoulos et al., 2013). For primers see Table S1. For GreenGate cloning, *BdMUTE* genomic region (BdiBd21-3.1G0240400) was amplified from Bd21-3 WT genomic DNA with priTN7+priTN8. The *ZmUBI* promoter and *BdMUTE* promoter were generated and used as previously described (Nunes et al., 2023). A backbone-specific GreenGate overhang was created in the process and the product was used to generate the respective GreenGate entry vector.

The entry modules, pGGD002 (D-dummy) and pGGE001 (*rbcS* terminator) are described in Lampropoulos et al. (2013) and pGGZ004 described in Lupanga et al. (2020). The entry module pGGB_3xGFP was generously provided by Jan Lohmann (Centre for Organismal Studies, Heidelberg).

The expression vectors *ZmUBIp:3xGFP-BdMUTE* and *BdMUTEp:3xGFP-BdMUTE* were assembled using the GreenGate cloning system. The six entry modules [pGGA_specific promoter (*MUTEp* or *ZmUBIp*); pGGB_3xGFP; pGGC_BdMUTE_genomic; pGGD_dummy; pGGE_terminator; pGGF_resistance; Zhang et al., 2022] were repeatedly digested and ligated with the destination vector pGGZ004 during 50 cycles (5 min 37°C followed by 5 min 16°C) followed by 5 min at 50°C and 5 min at 80°C for heat inactivation of the enzymes. All final constructs were test digested and the generated GreenGate overhangs were Sanger sequenced.

### Generation of transgenic lines

Generation of transgenic lines was performed as described in Zhang et al. (2022). In brief, embryonic calli were cultivated from isolated embryos from Bd21-3 and *sid* on callus induction media [CIM; per l: 4.43 g Linsmaier & Skoog media (LS; Duchefa), 30 g sucrose, 600 µl CuSO_4_ (1 mg/ml, Sigma/Merck), 500 µl 2,4-D (5 mg/ml in 1 M KOH, Sigma/Merck), pH 5.8, plus 2.3 g of Phytagel (Sigma/Merck)] for 3, 2 and 1 weeks, interspaced with transferral and splitting to fresh CIM media. Cultivation of the forming calli took place at 28°C, in the dark.

For transformation of the calli, AGL1 *Agrobacterium tumenfaciens* with the desired construct were scraped off selection plates and dissolved in liquid CIM media (same media as above without the Phytagel) with freshly added 2,4-D (2.5 mg/ml final concentration), Acetosyringone (200 mM final concentration, Sigma/Merck) and Synperonic PE/F68 (0.1% final concentration, Sigma/Merck). Around 100 calli (approximately two plates) were incubated in AGL1 in solution with OD600=0.6 for 15 min.

Afterwards, the calli were dried off on sterile filter paper, incubated for 3 days at room temperature in the dark and then moved to selection media [CIM+Hygromycin (40 mg/ml final concentration, Roche)+Timentin (200 mg/ml final concentration, Ticarcillin 2NA and Clavulanate Potassium, Duchefa)]. On selection media, the calli were incubated for 1 week and then moved to fresh selection plates and incubated for 2 weeks at 28°C in the dark. Next, the calli were moved to callus regeneration media [CRM; per l: 4.43 g of LS, 30 g maltose (Sigma/Merck), 600 µl CuSO_4_ (1 mg/ml), pH 5.8, plus 2.3 g of Phytagel, Timentin (200 mg/ml final concentration), Hygromycin (40 mg/ml final concentration) and sterile Kinetin solution (0.2 mg/ml final concentration, Sigma/Merck)]. The transformed calli were incubated at 28°C in a 16 h light/8 h dark cycle (PPFD 70-80 µmol m^−2^ s^−1^). Shoots >1 cm that formed after 2-6 weeks in the light on regenerating calli were transferred to rooting cups (Duchefa) containing rooting media [per l: 4.3 g Murashige & Skoog including vitamins (Duchefa), 30 g sucrose, pH 5.8, 2.3 g Phytagel, Timentin 200 mg/ml final concentration] and grown at 28°C in a 16 h light/8 h dark cycle (PPFD 70-80 µmol m^−2^ s^−1^). Once roots had formed, plants were moved to soil [four parts ED CL73 (Einheitserde), 1 part Vermiculite] and grown in a greenhouse/growth chamber with a 18 h light/6 h dark cycle (PPFD 250-350 µmol m^−2^ s^−1^).

### Microscopic analysis of SC and GC complementation phenotypes

Third leaves for quantitative assessment of rescue phenotype and for apex/middle ratio quantifications were collected from 3-week-old soil-grown plants. For adult leaf phenotyping, the leaf used for LI-6800 gas exchange measurements was collected. The leaves were fixed in 7:1 ethanol:acetic acid. Before imaging, leaf tissue was rinsed twice in water and mounted on slides in Hoyer's solution (Sharma, 2017). The abaxial side was imaged using a 40× objective on a Leica DM5000B or Leica DM2000 microscope with differential interference contrast (DIC) imaging.

3D-stacks of mature stomatal complexes were imaged on mature leaves of plants older than 5 weeks. Fixing and staining was adapted after Ursache et al. (2018) which in turn is based on Kurihara et al. (2015). The leaf tissue was fixed with 4% paraformaldehyde (PFA) in 1×PBS by pressure-injection with a needleless syringe and following 1 h incubation in 4% PFA with gentle agitation. The leaf was subsequently washed twice with 1× PBS and transferred to ClearSee solution {Xylitol [final concentration 10% (w/v)] (Sigma), sodium deoxycholate [final concentration 15% (w/v)] (Sigma), Urea [final concentration 25% (w/v)] (Sigma) in H_2_O}. The leaf tissue was cleared in ClearSee for at least 24-72 h with gentle agitation and stained with 0.1% Direct Red 23 (Sigma) in ClearSee overnight. To remove excess staining the leaves were washed in ClearSee for at least 1 h before imaging.

Imaging of 3D mature leaves was carried out using a Leica SP8 confocal microscope with 20% Argon laser, 561 nm excitation and detection at 580-615 nm at an intensity that yielded slight oversaturation for better assessment of the 3D structure. Gain was corrected in *z*-stacks using the *z*-compensation tool to acquire clear images in all *z*-planes. Stacks were imaged at resolution of 1024×1024 pixels, in 0.1 μm *z*-steps with a line average of 2.

### Microscopic analysis of reporter lines

For confocal imaging of reporter constructs, the developmental zone (first 2 mm) of the second leaf of 5-7 days post germination plate-germinated seedlings was used. Samples were stained with Propidium Iodide (PI; 10 μg/ml, Thermo Fisher Scientific) for ∼5 min to visualize cell walls, mounted in H_2_O on a slide and imaged with a 63× glycerol immersion objective on a Leica TCS SP8 microscope or a Leica Stellaris 5 microscope. On the SP8 we imaged using an Argon laser at 20% power, on the Stellaris 5 a white light LED at 85% laser power was used. At both microscopes, two high-sensitivity spectral detectors (HyD) were used to detect fluorescence emission (Leica Microsystems).

Laser intensity for fluorophore excitation was set to allow clear PI and fluorophore signals. Laser intensity was kept constant for quantification of the intensity, excitation of GFP with 488 nm at 15% intensity and 150% gain and PI excitation at 514 nm, adjusted for cell wall visibility. These settings allowed visualization of the lowest expressing 3xGFP line but led to saturation of the highest expressing line. The two YFP lines were excited with 12% intensity, 100% gain and plotted separately, as different fluorophores are not comparable. For all confocal microscopy, mid-plane images were used except when specified otherwise.

CTCF (El-Sharkawey, 2016) measurements were performed on confocal images of second leaf developmental zones. Images were acquired in stacks of 0.5 μm step size, 1024×1024 pixels and 2× line average. The average intensity of three adjoining slices with highest intensity of those stacks was taken to allow correction for nuclear position. Then the nucleus was traced with the Fiji polygon selection tool and the integrated density of the signal measured. We took 1-2 background measurements close to, but outside of, the nuclear signal. CTCF was calculated as: integrated density – (area of selected cell×mean fluorescence of background readings).

For mobility ratio measurements, images of M3GM++ and MYM were acquired on the Stellaris 5 confocal microscope with settings that allowed a visualization of SMC nuclear signal and GMC cytoplasmic signal. Those were 489 nm laser with 37.4% intensity and 200% signal gain for the 3xGFP line and 515 nm laser with 3.56% intensity and 75% signal gain for the YFP line. In addition, for the GFP line, the PI signal was excited with the 549 nm laser. When imaging YFP lines, PI was also excited with the 515 nm laser used to excite YFP. Images were acquired in stacks of four slices with 0.33 µm step size and 1024×1024 pixels. The average intensity of 2-3 slices of those stacks were taken to allow correction for nuclear position. Then a constant area was selected for each stomatal complex and the integrated density was measured for the SMC_(nuclear)_ and GMC_(cytoplasmic)_ signal. As a proxy for protein mobility, the ratio of SMC_(nuclear)_ and GMC_(cytoplasmic)_ signal was used. This also normalized for the difference in laser intensities.

Staging of the SC recruitment was performed on MYM and M3GM++ second leaf developmental zones. For staging relative to the first GC division, up to 15 stomatal complexes apically and basally of the first division in one stomatal row were analyzed to determine their GC division status (either yes or no) and their number of recruited SCs (zero, one or two). One stomatal cell file per individual was analyzed in a total of ten individuals.

SC recruitment linked to the LWR of the GMC/GC was also performed on MYM and M3GM++ second leaf developmental zones; here the length and width of the undivided or divided GMC was measured with the Fiji line tracing tool (perpendicular to the cell wall) and the complexes were analyzed regarding their GC division status (yes or no) and their number of recruited SCs (zero, one or two). A total of 129-191 stomatal complexes in two to six individuals were analyzed.

### Quantification of stomatal morphology

For quantification of the SC and GC division complementation phenotypes in WT, MYM, M3GM (++, +,− and −−), MMY and *sid*, 19-44 images of third leaves were acquired with the 40× objective at the DIC microscope. For five to twelve individuals, the stomatal phenotypes were counted in the four categories: 2 SC, 1 SC, 0 SC, aborted. Per individual, 74-244 stomatal complexes were counted.

GC morphology was quantified by measuring stomatal complexes of the M3GM− line flanked by zero, one or two SCs. The length of the GCs was measured along the longitudinal middle cell wall. Transverse GC width in the apices was measured at the widest and in the narrow central rod at the middle at the narrowest point. All measurements were performed with the line tracing tool in Fiji.

To correct for focal plane bias, 3D stacks of mature, Direct Red 23-stained stomatal complexes flanked by zero, one or two SCs of the M3GM− line were acquired. Of those complexes, *xz* optical cross-sections for each GC at three points of the *y*-axis and one *yz* optical section of each GC were taken. The *xz* optical cross-sections were made at the widest points of the apical and basal bulbous apices and the narrowest point of the middle rods. The area of the GC at these three sections was measured using the polygon selection tool in Fiji and the ratio of average apex area to middle area was taken as a proxy for GC dumbbell shape.

Quantifications of cell number and total tissue length between the first stage 2 cell (transverse asymmetric division) and the first stage 5 cells (longitudinal symmetric GMC division) comparing WT and *sid* were performed in developmental zones of plate-grown second leaves. Then 5-7 leaf zones per genotype were stained with PI and imaged using a 63× glycerol immersion objective on a SP8 confocal microscope (Leica Microsystems). Images were captured along the developmental zone of each sample starting at the first asymmetric division in the stomatal file and moving up to the first symmetric division of the GC precursors. Individual images along the stomatal rows were taken with at least 20% overlap and potential landmarks to later facilitate stitching and aligning. Images were processed with Fiji (Schindelin et al., 2012). Images were stitched pairwise using the ‘stitching’ plugin and manually whenever pairwise stitching was not possible. To quantify the cell number between developmental zones, cells were counted using the multi-point tool and the distances between first ACD and GCD calculated.

Division orientation of GMCs in *sid*, MMY, M3GM−− and WT was assessed using confocal imaging. Images were obtained of PI-stained developmental zones from second leaves of plate-germinated seedlings using the 63× objective. Both the imaging and the analysis was carried out fully anonymized (i.e. neither the person doing the microscopy nor the person doing the analysis knew the genotype of the sample). A straight line was drawn connecting the two apical or the two basal lateral corners of the divided GMC of the stomatal complex. Then, the left and right distances were measured as distance from the corner to the intersecting GMC division plane on the previously drawn line (Fig. 4B). For each stomatal complex, the smaller distance was divided by the bigger distance to obtain the ratio of the division plane skewness.

All image analysis and processing was carried out in Fiji (Schindelin et al., 2012). Images shown in Figs 1, 3, 4 and 5 were processed with background reduction (rolling ball 50) and brightness and contrast adjustments. For pictures shown in Fig. 2 only the PI channel was adjusted. No post-processing was performed on images that were used for quantification.

### RNA fluorescence *in situ* hybridization

Whole-mount RNA fluorescence *in situ* hybridization of leaf developmental zones of 3-week-old WT plants was conducted using the HCRTM RNA-FISH system (Molecular Instruments) (Dirks and Pierce, 2004; Choi et al., 2018). The protocol used here was adapted from Huang et al. (2023) and Chelysheva et al. (2024) and spans 5 days from material collection to final confocal imaging. On day 1, young, not yet unrolled, leaves were collected and the bottom 5-8 mm was harvested in a Petri dish in a few drops of a fixative solution {Fixative FAA; formaldehyde solution [final concentration 4% (v/v)] (Sigma), glacial acetic acid [final concentration 5% (v/v)] (Fisher Scientific), absolute ethanol [final concentration 50% (v/v)] in nuclease-free water (Thermo Fisher Scientific)}. The leaf piece was cut once in longitudinal and once in transverse direction and then collected in a 2 ml tube with Fixative FAA and this was repeated for four to five additional leaves that were added to the same sample tube. After sample collection, vacuum was applied to the tube several times until all leaf pieces were submerged and then incubated at room temperature (RT) for 3 h. Fixative FAA was replaced by a series of ethanol concentrations (10%, 30%, 50%, 70%) and, for each concentration, the samples were microwaved five times for 30 s at 180 W (Chelysheva et al., 2024). After the last microwaving step with 70% ethanol, the sample was stored in a −20°C freezer (the sample can be stored like this for several weeks).

On day 2, the sample was allowed to warm up to RT and then rehydrated through a series of washes at RT on a tube revolver (Thermo Fisher Scientific): first with 50% ethanol/50% DPBS-T {Tween20 [final concentration 0.1% (v/v)] (Sigma) in Dulbecco's phosphate-buffered saline (DPBS) (Gibco)} for 15 min and then twice with 100% DPBS-T for 15 min each step. Aliquots of Proteinase K solution [1 M Tris-HCl, pH 8 (final concentration 0.1 M), 0.5 M EDTA, pH 8 (final concentration 0.05 M), Proteinase K (final concentration 80 µg/ml) (Thermo Fisher Scientific) in nuclease-free water] had been prepared and frozen at −20°C and one aliquot was taken out to thaw at RT during the first incubation step with 100% DPBS-T. The DPBS-T was replaced by the Proteinase K solution and incubated by applying vacuum for 5 min and then digesting for 25 min at 37°C on the thermomixer (Eppendorf). During this step, the sample was agitated every 5 to 10 min. Subsequently, the sample was washed twice for 15 min in DPBS-T at RT on the tube rotator. The second fixative solution {Fixative II; formaldehyde solution [final concentration 4% (v/v)] in DPBS-T} replaced the DPBS-T and the sample was again vacuum-infiltrated for 10 min followed by 20 min at RT on the tube rotator. In the meantime, two 500 µl aliquots of the HCRTM Probe Hybridization Buffer (Molecular Instruments) were prepared: one was left to reach RT and the other was put to 37°C in the thermomixer. The sample was washed twice for 15 min in DPBS-T at RT on the tube rotator and then incubated in the RT aliquot of the Probe Hybridization Buffer by applying vacuum for 10 min and then pre-hybridized for 1 h at 37°C in the thermomixer with shaking (1000 rpm). The probe solution was prepared by adding 0.8 pmol (i.e. 2 µl of the 1 µM stock) of each HCRTM probe (designed and created by Molecular Instruments for the *bdmute* gene) to 500 µl of the Probe Hybridization Buffer at 37°C. As much as possible was removed from the pre-hybridization solution while being careful to not pick up the leaf pieces and the final probe solution was added to the sample before an overnight (22 h) incubation step at 37°C in the thermomixer with shaking (1000 rpm).

To prepare for day 3, an aliquot of HCRTM Probe Wash Buffer (Molecular Instruments) was warmed up to 37°C. Additionally, two aliquots of HCRTM Amplification Buffer were prepared and warmed up to RT: one with 250 µl and one with 500 µl. The excess probes were washed out by washing four times with 500 µl Probe Wash Buffer for 15 min at 37°C in the thermomixer with shaking (1000 rpm). Another two washing steps with SSC-T buffer {20× SSC buffer [final concentration 25% (v/v)] (Invitrogen), Tween20 [final concentration 0.1% (v/v)] in nuclease-free water} for 5 min each at RT in the thermomixer with shaking (1000 rpm) were carried out and the rest of the SSC-T buffer was stored at 4°C. Next, the hairpins h1 and h2 for the amplifier B1 with fluorophore 488 (Molecular Instruments) were put in an ice bucket to slowly thaw. In the meantime, the SSC-T buffer was replaced by 500 µl Amplification Buffer and vacuum was applied for 10 min before 50 min pre-amplification at RT on the tube rotator. During this incubation step, 6 pmol of hairpins h1 and h2 (i.e. 5 µl of the 3 µM stocks) were put in separate tubes, heated at 95°C for 90 s and then kept in a dark drawer at RT for 30 min. Then they were assembled in the prepared tube of 250 µl Amplification Buffer. As much as possible of the pre-amplification solution was removed while trying to avoid damage to the leaf pieces and then the Amplification Buffer with hairpins h1 and h2 was added to the sample and incubated for about 42 h in the dark at RT.

On day 5, the SSC-T buffer was taken from the 4°C fridge to warm up to RT. Excess hairpins were removed from the sample by washing with SSC-T buffer in a series of washes: twice for 5 min, twice for 30 min and once for 5 min. To visualize cell walls, the sample was stained with SCRI Renaissance Stain 2200 {SR2200, Tokyo Future Style, Inc. [final concentration 0.001% (v/v)] in SSC-T buffer} for 1 min and then washed three times with SSC-T buffer. For the imaging, a Leica Stellaris 5 was used with the following settings: SR2200 was imaged with 405 nm excitation and 414-442 nm emission at 1% laser intensity and 50% gain. The BdMUTE probe signal was imaged with 490 nm excitation and 507-532 nm emission at 10% laser intensity and 100% gain. Images for Fig. S3 were analyzed, and brightness and contrast were adjusted in Fiji.

### Quantification of stomatal physiological responses

The LI-6800 Portable Photosynthesis System (LI-COR Biosciences Inc) infrared gas analyzer was used to obtain leaf level gas exchange measurements. As described in Nunes et al. (2022), the youngest, fully expanded leaves of 3- to 4-week-old soil-grown plants were measured using the following conditions in the chamber system: flow rate, 500 µmol s^−1^; fan speed, 10,000 rpm; leaf temperature, 28°C; relative humidity (RH), 40%; [CO_2_], 400 µmol mol^−1^. PPFD 1000, 100, 1000, 0 µmol m^−2^ s^−1^, 20 min per step. Every 60 s the system automatically logged gas exchange and environment conditions including absolute stomatal conductance (*g*_sw_) and carbon assimilation (*A*). In total 26 WT individuals due to repeated paired experiments and four to six individuals for the M3GM lines (++, +, − and −−), MYM, MMY and *sid* were used and the respective leaf section that was inside the Li-6800 chamber was collected in 7:1 ethanol:acetic acid for fixation and clearing for subsequent microscopy (see above). All measurements were corrected in the respective LI-6800 Excel output file by individual leaf area calculated as leaf width multiplied by the chamber diameter. The Excel files were used as input for the licornetics R package (version 2.1.2; https://github.com/lbmountain/licornetics) to average the data across individuals, divide the values by stomatal density to obtain values per stoma and visualize it. To quantify the complementation phenotypes, 9-10 DIC images per individual were taken using the 40× objective. Phenotypes were categorized as ‘2 SCs’, ‘1 SC’, ‘0 SC’ and ‘aborted’ (see above). These images were also used to calculate stomatal densities (stomata per mm^2^) and physiological parameters were corrected by mean stomatal density per line in the licornetics package to obtain values per stoma.

### Statistical analysis and data visualization

Downstream analysis of spreadsheet data files and visualization was conducted in R Studio (version 2023.12.1; https://posit.co/products/open-source/rstudio/) using R (version 4.3.1; R-project.org). Statistical analysis was carried out using either unpaired, two-sided Student's *t*-test or one-way ANOVA (stats package version 4.3.1; R-project.org) and Tukey's HSD test (Agricolae package version 1.3-6; https://rdrr.io/cran/agricolae/). Further packages required for file management, calculations and final visualization were: readxl (version 1.4.3; https://readxl.tidyverse.org/), tidyverse (version 2.0.0; Wickham et al., 2019), ggpubr (version 0.6.0; https://rpkgs.datanovia.com/ggpubr/), ggtext (version 0.1.2; https://cran.r-project.org/web/packages/ggtext/) and MetBrewer (version 0.2.0; https://github.com/BlakeRMills/MetBrewer).

## Supplementary Material



10.1242/develop.203011_sup1 Supplementary information
