# Supplementary material for: Dual role of BdMUTE during stomatal development in the model grass Brachypodium distachyon
Source: Development. 2024 Sep 26;151(20):dev203011. doi: 10.1242/dev.203011 (PMC11449446; doi:10.1242/dev.203011)
Supplement: Supplementary information [file develop-151-203011-s1.pdf]

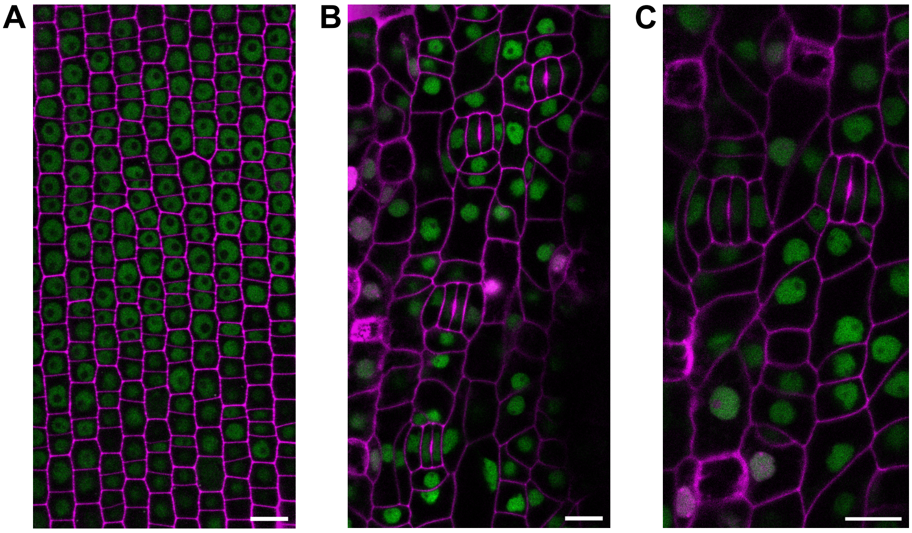

Fig. S1. Ubiquitously expressed 3xGFP-BdMUTE in wild type (WT) background (WT*;ZmUBIp:3xGFP-MUTE*) induces ectopic divisions. (**A)** Early developmental zone of the leaf epidermis shows expression of 3xGFP-MUTE (green) in all cells. **(B, C)** Later stages of epidermal development show ectopic, subsidiary cell-like divisions. Shown are midplane confocal images of developmental zones in T0 lines stained with propidium iodide (PI, magenta). Scale bars = 10 μm.

Development • Supplementary information

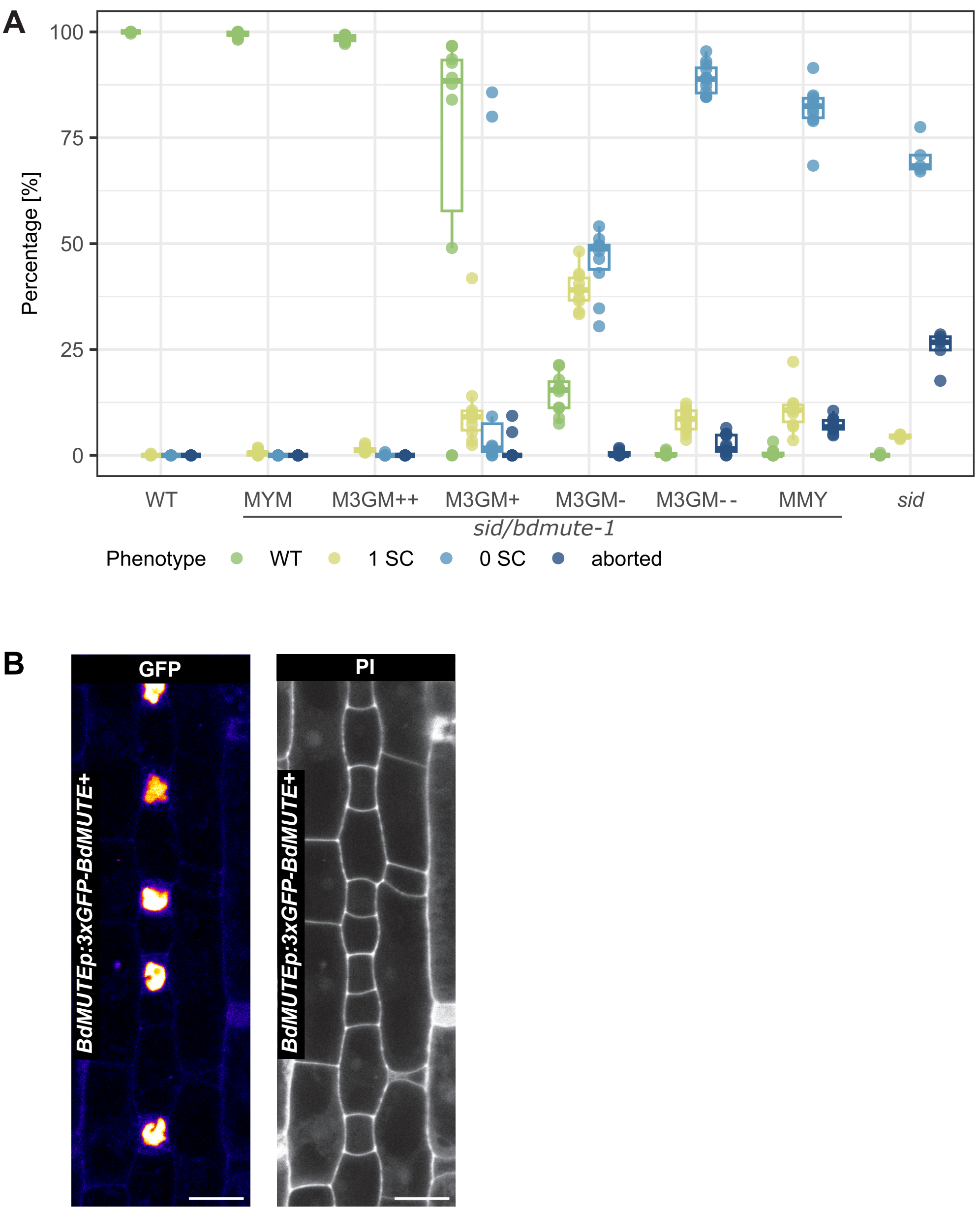

Fig. S2. (A) Complementation of *sid/bdmute-1* stomatal phenotypes by different complementation lines (same data as in Fig. 2E). Plant lines are wild type (WT), *sid/ bdmute-1*;*BdMUTEp:YFP-BdMUTE* (MYM), four *sid/bdmute-1*;*BdMUTEp:3xGFP-BdMUTE* lines (M3GM++, M3GM+, M3GM- and M3GM- -), *sid/bdmute-1*;*BdMUTEp: BdMUTE-YFP* (MMY) and *sid/bdmute-1*. Data from fully expanded 3rd leaves of soil-grown plants 19-21 days after germination. Boxplots of data shown in Fig. 2E. n = 5-12 individuals per genotype and 864-1450 stomata per genotype. Each dot represents one individual. (B) Confocal image of the M3GM+ line shown in Fig. 2D here shown as split channels to resolve that SMC nuclear signal is noise from the propidium iodide (PI) channel. Signal intensity of reporter proteins shown as “fire” heatmap and PI-stained cell walls shown in gray. GFP was imaged with 15% laser and 150% gain. Scale bars = 10 µm.

Development • Supplementary information

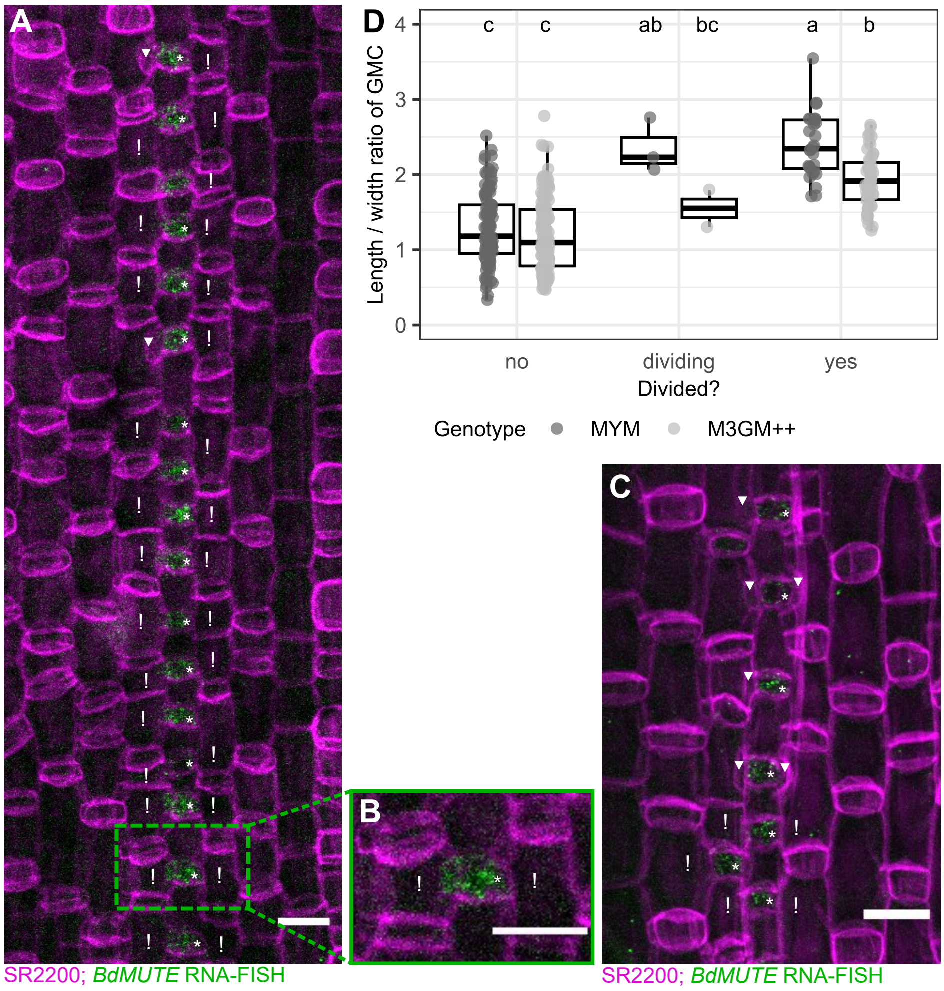

Fig. S3. (A, B, C) *BdMUTE* mRNA-fluorescence in situ hybridization (RNA-FISH). Shown are confocal images of leaf epidermis developmental stages 3-4 with BdMUTE probe signal (green) and SR2200-stained cells (magenta). Asterisks (*) indicate guard mother cells (GMCs), exclamation points mark the subsidiary mother cells (SMCs), and arrowheads point to divided subsidiary cells (SCs). (B) shows a close up of a GMC from (A) with signal visible in the GMC and only background signal visible in the neighbouring SMCs. Scale bars = 10 µm. (D) LWRs of *sid/bdmute-1*;*Bd-MUTEp:YFP-BdMUTE* (MYM) and *sid/bdmute-1*;*BdMUTEp:3xGFP-BdMUTE* (M3GM++) guard cell complexes analyzed in Fig. 3E before, during and after GMC division. n = 2-6 individuals per genotype and 129-191 stomata per genotype. Each dot represents one stomatal complex. Signifi-cant differences are indicated with differing letters. Statistical test: ANOVA followed by Tukey's HSD test (alpha = 0.05).

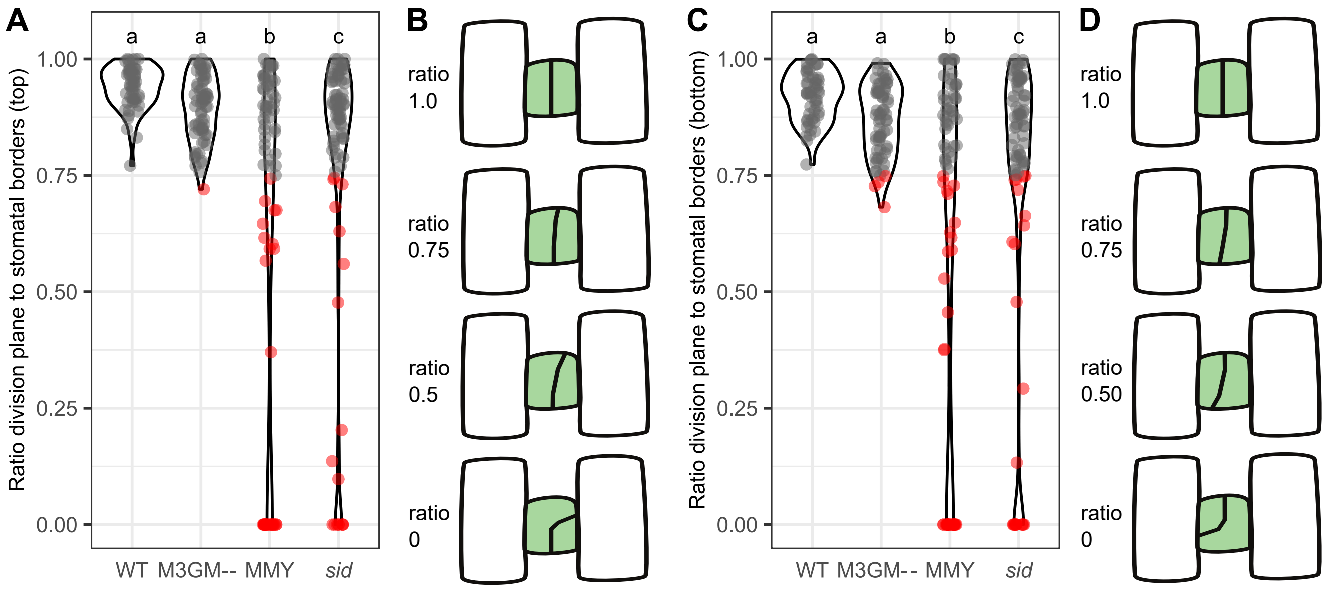

Fig. S4. "Skewness" ratios of division plane orientation symmetry at the top and bottom of the guard mother cells (GMCs) in wild type (WT), *sid/bdmute-1*;*BdMUTEp:3xGFP-BdMUTE* (M3GM- -), *sid/bdmute-1*;*BdMUTEp:BdMUTE-YFP* (MMY) and *sid/bdmute-1*. Combined "skewness" ratios per stomatal complex are depicted in Fig. 4C. A straight line was drawn to connect the outward corners of the GMC at the top and bottom respec-tively (yellow line in Fig. 4B) and the distance from each corner to the intersection with the GMC division plane was measured along the previously drawn line (pink lines in Fig. 4B). The smaller distance was divided by the longer distance to obtain the ratio of division plane to stomatal borders plotted in **(A)** for the apical wall of the GMC. A ratio of 1 means the GMC divided longitudinally along the central plane, a ratio below 1 indicates a more skewed division orientation and at a ratio of 0 the division plane did not cross the upper stomatal border (i. e. transversal division). Gray dots indicate a ratio > 0.75 and red dots a ratio < 0.75. **(B)** Schematic representation of symmetric or skewed division plane orientation at the apical wall of the GMC with the respective ratios of division plane to stomatal borders. **(C)** Ratio of division plane to stomatal borders at the basal wall of the GMC. A ratio of 1 means the GMC divided longitudinally along the central plane, a ratio below 1 indicates a more skewed division orientation and at a ratio of 0 the division plane did not cross the lower stomatal border (i. e. transversal division). Gray dots indicate a ratio > 0.75 and red dots a ratio < 0.75. **(D)** Schematic representation of symmetric or skewed division plane orientation at the basal wall of the GMC with the respective ratios of division plane to stomatal borders. n = 7-8 individuals per genotype and 66-82 stomata per genotype (dots are stomata). Significant differences are indicated with differing letters. Statistical test: ANOVA followed by Tukey's HSD test (alpha = 0.05).

Development • Supplementary information

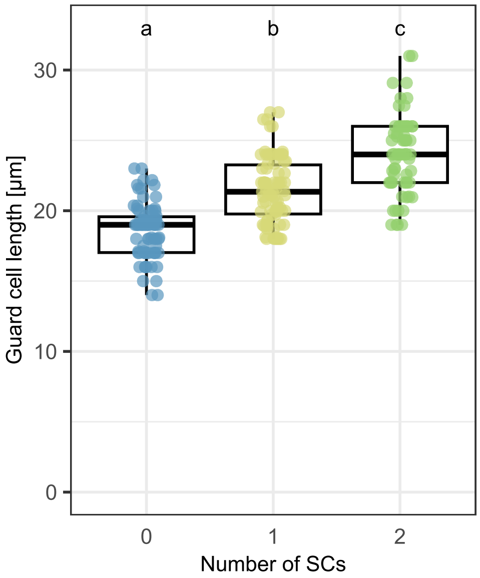

Fig. S5. Guard cell length in *sid/bdmute-1*;*BdMUTEp:3xGFP-BdMUTE* (M3GM-) complexes with zero, one or two subsidiary cells (SCs). Same cells as measured in Fig. 5B. n = 2 individuals and 16-36 stomata per phenotype. Each dot represents one stomatal complex. Significant differenc-es are indicated with differing letters. Statistical test: ANOVA followed by Tukey's HSD test (alpha = 0.05).

Development • Supplementary information

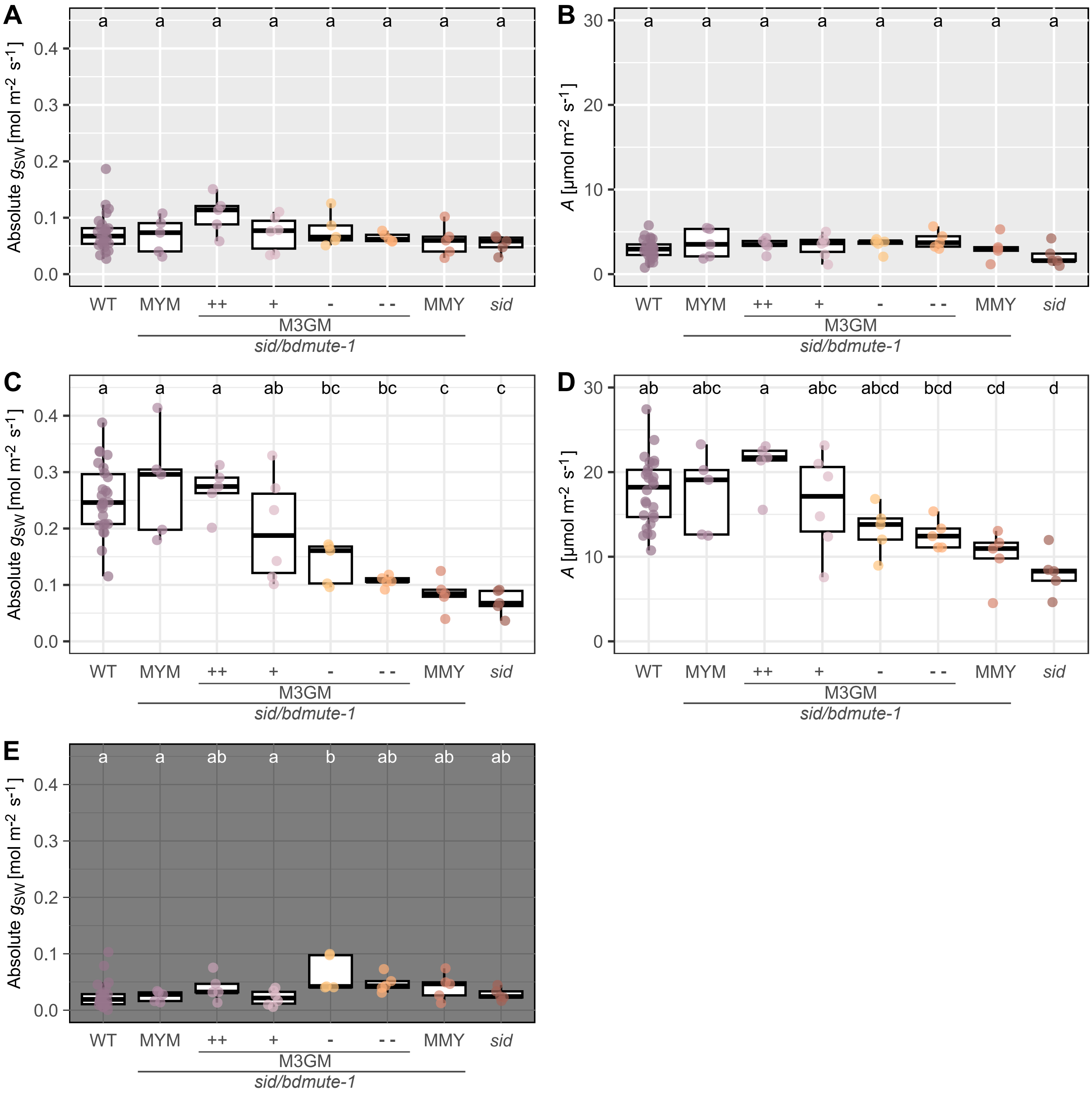

Fig. S6. Steady state gas exchange values of measurements shown in Fig. 6 for wild type (WT), *sid/bdmute-1*;*BdMUTEp:YFP-BdMUTE* (MYM), the four different *sid/ bdmute-1*;*BdMUTEp:3xGFP-BdMUTE* (M3GM++ to - -) lines, *sid/bdmute-1*;*BdMUTEp: BdMUTE-YFP* (MMY) and *sid/bdmute-1*. Average value of 5 minutes at the end of each light intensity step. Corresponding light intensity step is indicated by background color: Light gray (100 µmol m^{-2} s^{-1}), white (1000 µmol m^{-2} s^{-1}), and dark gray (0 µmol m^{-2} s^{-1}). **(A) (C) (E)** Absolute stomatal conductance (g_{SW}). **(B) (D)** Carbon as-similation (A). n = 5-6 individuals per genotype, 26 individuals for WT. Dots represent individuals. Significant differences are indicated with differing letters. Statistical test: ANOVA followed by Tukey's HSD test (alpha = 0.05).

Development • Supplementary information

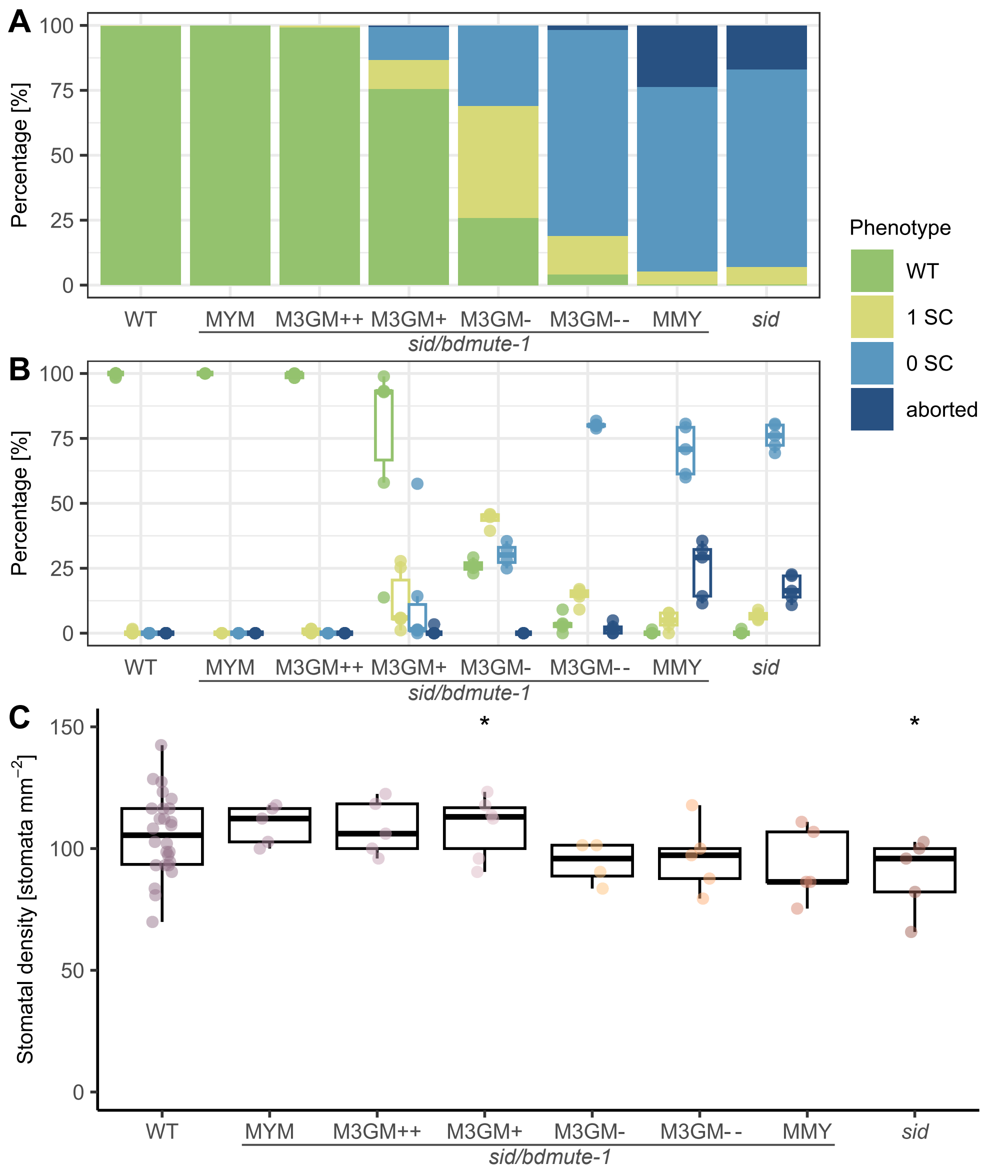

Fig. S7. Stomatal morphology complementation phenotypes and stomatal density of leaves used for the leaf-level gas exchange measurements (Fig. 6) of wild type (WT), *sid/ bdmute-1*;*BdMUTEp:YFP-BdMUTE* (MYM), the four different *sid/bdmute-1*;*BdMUTEp: 3xGFP-BdMUTE* (M3GM++ to - -) lines, *sid/bdmute-1*;*BdMUTEp:BdMUTE-YFP* (MMY) and *sid/bdmute-1*. **(A)** Stacked bar plot of complementation of the *sid/bdmute-1* background in adult leaf. "Aborted" describes complexes with oblique GMC division or aborted development. **(B)** Same data as in (A) depicted as box- and dot plot with dots representing individuals; n = 4-6 individuals per genotype, 26 individuals for WT. **(C)** Stomatal densities of the different lines; dots represent individuals. Asterisk indicates a significant difference to the WT individuals measured within the same experiment. n = 4-6 individuals per genotype, 26 individuals for WT, and 266-502 stomata per genotype. Statistical test: Two-sided Student's t-test (significant if $p < 0.05$) of the respec-tive genotype compared to the WT individuals (n = 4-6) grown at the same time.

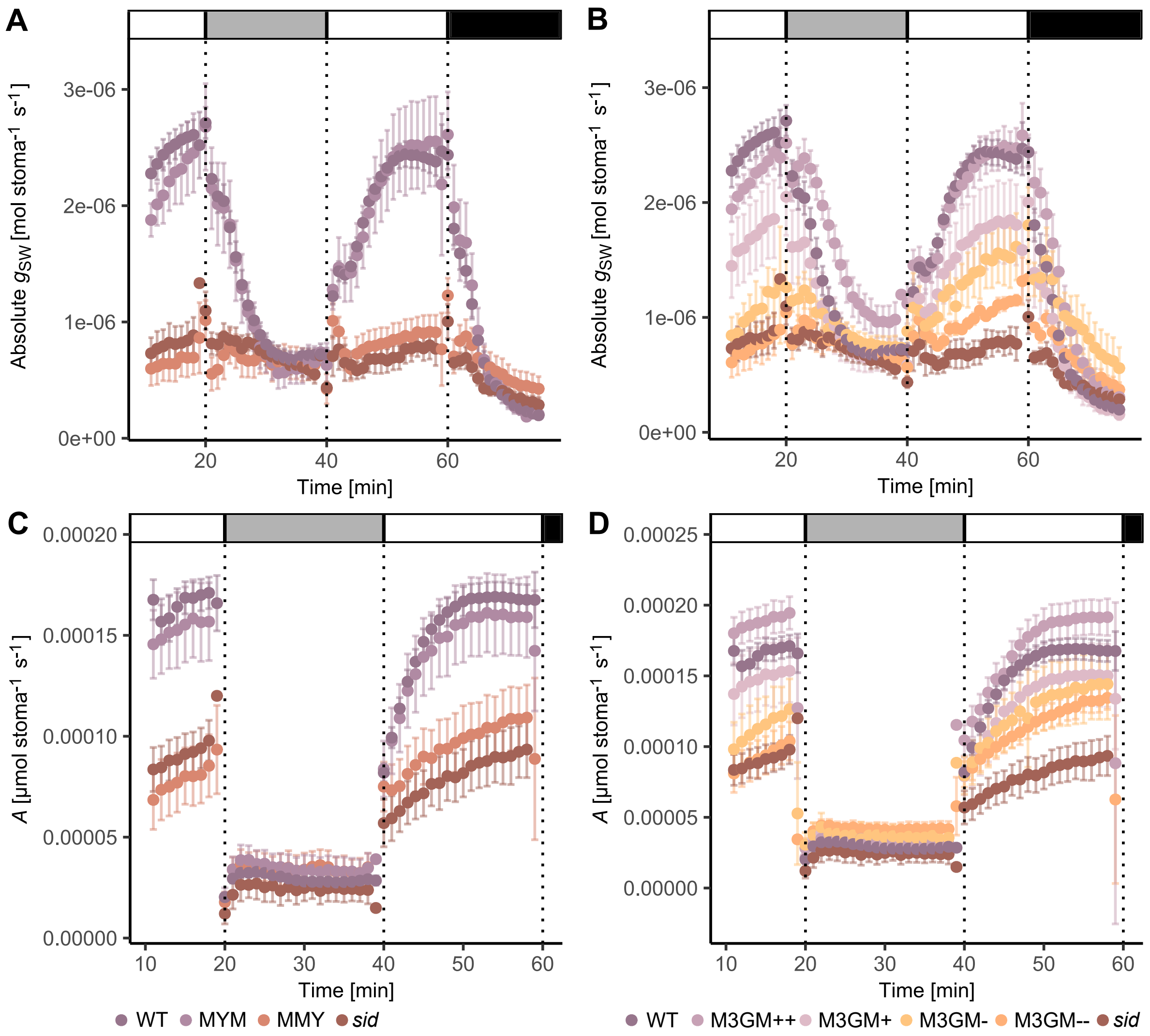

Fig. S8. Leaf-level gas exchange values per stoma under changing light conditions. Data is the same as shown in Fig. 6 corrected by mean sto-matal density per genotype. Changing light conditions are indicated by grayscale bars above each plot: White = 1000 µmol m^{-2} s^{-1}, light gray = 100 µmol m^{-2} s^{-1}, black = 0 µmol m^{-2} s^{-1}. **(A)** Absolute stomatal conductance (g_{SW}) of wild type (WT), *sid/bdmute-1*;*BdMUTEp:YFP-BdMUTE* (MYM), *sid/ bdmute-1*;*BdMUTEp:BdMUTE-YFP* (MMY) and *sid/bdmute-1*. **(B)** g_{SW} of WT, the four different *sid/bdmute-1*;*BdMUTEp:3xGFP-BdMUTE* (M3GM) lines (++, +, - and - -) and *sid/ bdmute-1*. **(C)** Carbon assimilation (*A*) of WT, MYM, MMY and *sid/bdmute-1*. **(D)** *A* of WT, M3GM lines and *sid/bd-mute-1*. Note that the same WT and *sid/bdmute-1* data is shown in A, C and B, D. Measured were the youngest, fully expanded leaves of 3-4 week old, soil-grown plants; n = 5-6 individuals per genotype, 26 individuals for WT. Dots are means averaged across the individuals with error bars indi-cating standard error.

Table S1. Primers for Greengate cloning of reporter constructs

name	sequence	comments
priTN1	AACAGGTCTCA**ACCT***CAG GCTAGCAGCACTATT*	FP amplifying BdMUTEpromoter with **pGGA**000 BsaI sites (~priJM_BdMUTEproFW)
priTN2	AACAGGTCTCA**TGTT***GATC GTGTCGTTCTTC*	RP amplifying BdMUTEpromoter with **pGGA**000 BsaI sites (~*priJM_BdMUTEproREV*)
priTN3	AACAGGTCTCA**ACCT***GAA ACTTCACGATCGGCTCT*	FP amplifying ZmUBI1 promoter with **pGGA**000 BsaI sites (~priJM_ZmUBI1proFW)
priTN4	AACAGGTCTCA**TGTT***GAC CTGCAGAAGTAACACCA*	RP amplifying ZmUBI1 promoter with **pGGA**000 BsaI sites (~priJM_ZmUBI1proREV)
priTN7	*AACAGGTCTCA***GGCTCT***A TGTCGCACATCGC*	FP amplifying BdMUTE ORF with **pGGC**000 BsaI sites (~priJM_BdMUTEORF_FW)
priTN8	*AACAGGTCTCA***CTGA***TTAA TTGATCATGATGTCGCCAT*	RP amplifying BdMUTE ORF with **pGGC**000 BsaI sites (~primr265_BdMUTEORF_REV)
priTN9	AACAGGTCTCA**ACTA**GAA ACTTCACGATCGGCTCT	FP amplifyingHygR cassette (ZmUBI1p driven) with **pGGF**000 BsaI sites (~priJM_HygRC_ZmUBI1_FW)
priTN10	AACAGGTCTCA**ATAC**CCT GCAGGTCACTGGATTTT	RP amplifyingHygR cassette (ZmUBI1p driven) with **pGGF**000 BsaI sites (~priJM_HygRC_ZmUBI1_REV)
priTN11	AACAGGTCTCA**ACTA**AGA AGCCAACTAAACAAGACC A	FP amplifyingHygR cassette (PvUBI2p driven) with **pGGF**000 BsaI sites (~priJM_HygRC_PvUBI2_FW)
priTN12	AACAGGTCTCA**ATAC**TTAA TTCGGGGGATCTGGATTT	RP amplifyingHygR cassette (PvUBI2p driven) with **pGGF**000 BsaI sites (~priJM_HygRC_PvUBI2_REV)
priTN13	AACAGGTCTCA**ACTA**ACTA GATCCCATGAGCTTGC	FP nptII with **pGGF**000 BsaI sites (~priJM_nptIIFW)
priTN14	AACAGGTCTCA**ATAC**GGT TTTAACCGTACGCGTTT	RP nptII with **pGGF**000 BsaI sites (~priJM_nptIIREV)

Development • Supplementary information
