# Supplementary material for: Dual role of BdMUTE during stomatal development in the model grass Brachypodium distachyon
Source: Development. 2024 Sep 26;151(20):dev203011. doi: 10.1242/dev.203011 (PMC11449446; doi:10.1242/dev.203011)
Supplement: Supplementary file 1 [file develop-151-203011_review_history.pdf]

Dual role of BdMUTE during stomatal development in the model grass *Brachypodium distachyon*

Roxane P. Spiegelhalder, Lea S. Berg, Tiago D. G. Nunes, Melanie Dörr, Barbara Jesenofsky, Heike Lindner and Michael T. Raissig
DOI: 10.1242/dev.203011

Editor: Liam Dolan

Review timeline

Original submission:	30 April 2024
Editorial decision:	31 May 2024
First revision received:	7 August 2024
Accepted:	12 August 2024

Original submission

First decision letter

MS ID#: DEVELOP/2024/203011

MS TITLE: Dual role of BdMUTE during stomatal development in the model grass *Brachypodium distachyon*

AUTHORS: Roxane P Spiegelhalder, Lea S Berg, Tiago DG Nunes, Melanie Dörr, Barbara Jesenofsky, Heike Lindner, and Michael T Raissig

I have now received all the referees reports on the above manuscript, and have reached a decision. The referees' comments are appended below, or you can access them online: please go to BenchPress and click on the 'Manuscripts with Decisions' queue in the Author Area.

The overall evaluation is positive and we would like to publish a revised manuscript in Development, provided that the referees' comments can be satisfactorily addressed. Please attend to all of the reviewers' comments in your revised manuscript and detail them in your point-by-point response. If you do not agree with any of their criticisms or suggestions explain clearly why this is so. If it would be helpful, you are welcome to contact us to discuss your revision in greater detail. Please send us a point-by-point response indicating your plans for addressing the refereesâ€™ comments, and we will look over this and provide further guidance.

Reviewer 1

Advance summary and potential significance to field

Grasses develop a unique stomatal structure that consists of two dumbbell-shaped guard cells flanked by two lateral subsidiary cells. Recent work has revealed that BdMUTE, which encodes a bHLH protein, recruits SCs and regulates GMC fate and GC morphogenesis (Raissig et al., 2017). It is also known that BdMUTE is expressed in the GMC and that the protein encoded by this gene moves from the GMC to epidermal cells of neighbouring files. Although several observations have allowed us to delve into the cell-autonomous and cell non-autonomous signalling mediated by BdMUTE (Serna, 2020 and 2021), direct proofs conclusively validating these ideas are lacking. By analysing four lines with severe expression variability of a mobile-reduced, but functional BdMUTE construct

© 2024. Published by The Company of Biologists under the terms of the Creative Commons Attribution License (https://creativecommons.org/licenses/by/4.0/).

(BdMUTEp:3xGFP-BdMUTE) in sid/bdmute-1, Spiegelhalder and co-workers experimentally address the signalling through BdMUTE during stomatal development.

The main findings are:

1) BdMUTEp:3xGFP-BdMUTE rescues the formation of SCs of sid/bdmute-1 in a dose-dependent manner, but the order of GMC and SC divisions, where the SC divisions always precede the GMC divisions, is disturbed, delaying SC recruitment. This is telling us that BdMUTE mobility is required non-cell autonomously to avoid a delay in SMC divisions.

2) BdMUTEp:3xGFP-BdMUTE rescues the GMC divisions of sid/bdmute-1 also in a dose-dependent manner. In addition, weak BdMUTEp:3xGFP-BdMUTE expression in the GMC rescues GMC division irrespective of SC recruitment. Therefore, the role of BdMUTE in triggering GMC division does not require SC formation.

3) A BdMUTEp:3xGFP-BdMUTE line (M3GM-) that exhibited almost equal number of stomatal complexes with zero, one or two SCs, shows that SCs formation is not required for BdMUTE genes to trigger, in a non-cell autonomously manner, GC morphogenesis.

4) BdMUTEp:3xGFP-BdMUTE rescues the stomatal physiological defects of sid/bdmute-1 in a dose-dependent manner, which mirrors the developmental complementation gradient mediated by distinct levels of BdMUTEp:3xGFP-BdMUTE.

Comments for the author

In essence, this manuscript provides an interesting set of data that illuminates the role of BdMUTE during stomatal complexes development. My main criticism concerns the assignment of a GMC-autonomous function during GMC division to BdMUTE. In Fig 2 (see also Results, Section 1), the authors indicate that “mobile signal in the lateral cell files is only visible in the single-tag YFP”. Certainly, this signal is seen in SMCs of these lines, but it can also be visualized in SMCs of M3GM+ line (Fig. 2 D). In Fig 3, the signal can be also visualized in SMCs of M3GM++. Because the assigned GMC-autonomous function of BbMUTE rests on the absence of BbMUTE protein in SMCs, the authors should reconsider this point. Could lines M3GM- and/or M3GM-- be used to establish that BdMUTE acts cell-autonomously in guiding GMC division?

Minor points:
1) PP3, lines 51 and 52: “Grass SC are …” should be supported by a reference.
2) PP3 and 4, lines 68-74: The role of the Arabidopsis bHLH should be succinctly described.
3) Fig. 2: The term “aborted” should be defined (“GMC dividing oblique and/or aborted”).
4) PP13, line 375, “has a GC-autonomous role during GC division…” should be replaced by “has an autonomous role during GMC division…”
5) PP15, line 460, “GC” should be replaced by “GMC”.
6) PP16, line 494, “SC divisions” should be replaced by “SMC divisions”.
7) Fig 3D: “Divided GMC?” should be replaced by “Divided GMC”.
8) PP16, line 498: “guard cell division orientation” should be replaced by “guard mother cell division orientation”.
9) PP16, line 508, “GC and SC divisions” should be replaced by “GMC and SC divisions”.

My best wishes,

Reviewer 2

Advance summary and potential significance to field

Stomatal regulator BdMUTE is expressed in guard mother cells (GMCs) and the protein was reported to move laterally into subsidiary mother cells (SMCs) to induce asymmetric divisions and subsidiary cell (SC) formation in the model grass Brachypodium (Raissig et al. 2017). Spiegelhalder et al. generated a mobility impaired 3xGFP-BdMUTE fusion protein to disentangle the potential role of BdMUTE in guard mother cells (where it is expressed) vs. its role in SMCs (where it moves to). This

© 2024. Published by The Company of Biologists under the terms of the Creative Commons Attribution License (https://creativecommons.org/licenses/by/4.0/).

approach seems suitable to study the hypothesis that BdMUTE has distinct roles in two different cell types. Moreover, the BdMUTE system allows to study how intercellular mobility of regulators affects cell fate regulation in plants in general and is therefore of interest to a broader audience. The authors found that - depending on protein dosage- 3xGFP-BdMUTE could still fully rescue subsidiary cell development in the sid/bdmute mutant background (strongest expressing line) or barely do so (weakest line). The only difference between the control YFP-BdMUTE and the strongest 3xGFP-BdMUTE line was a temporal delay in SC recruitment in the latter. However, even the weak 3xGFP-BdMUTE line could induce symmetric cell divisions in guard cells, which indicates that BdMUTE also acts locally on promoting guard cell fate in GMCs, a role that does not require BdMUTE mobility and SC recruitment. These results are very nice and indeed suggest a dual role for BdMUTE in SC and GC formation.

Comments for the author

I fully support the hypothesis and the approach authors have taken in this study and would like to emphasise the tremendous amount of well-executed statistical analyses presented here, which is quite impressive - although it sometimes even distracts the reader from the main story. The manuscript is very lengthy and that made it partially confusing for me. Also some of the assumptions (e.g. that the BdMUTE protein is mobile) are not sufficiently explained and backed up by evidence in my opinion.

Here are some detailed suggestions and requests:

Major points:
1) This manuscript is based on the assumption that the BdMUTE protein is both mobile and functional as long as fusion proteins are only fused to its N-terminus. Hence, a bigger N-terminal fusion protein, such as 3xGFP should just impair mobility but not function. Unfortunately, 3xGFP did not render BdMUTE immobile and only delayed its migration to and activity in SMCs. Could the authors please elaborate why they assume that the protein and not the BdMUTE mRNA is mobile? If the mRNA is transported into SMCs and translated there, it could explain why this approach with 3xGFP tag did not work as expected. There could be something particular about the 3' end of the BdMUTE mRNA that allows migration to other cells or affects its stability while being transported. Altered mRNA stability in M3GM++ during migration could also account for the lower ratio between nuclear SC and cytoplasmic GMC intensity (Figure 3B). If the authors think that a detailed analysis of how BdMUTE mobility works is beyond the scope here, I can understand, but some evidence should be presented or discussed (if already published elsewhere) that the protein is the mobile part.

2) Since 3xGFP-BdMUTE showed dose-dependent effects, I am missing a control of YFP-BdMUTE (MYM) at lower concentration levels. MYM seems to be a quite strong line and intensity-wise just like M3GM++ (Figure 2 and Figure 3A). Even the addition of one weak MYM line would be enough to show that the 3xGFP tag delays mobility. Without that control, I do not think that impaired mobility can be assumed for M3GM++.

3) Overall, the authors present a quite impressive number of statistical analyses (in 6 main figures and 10 supplementary figures) that all seem well executed and conclusive but do not necessarily address the core question about local vs. mobile BdMUTE function they set out to answer. Figure 5 and 6 almost feel like a different story. The effect of subsidiary cells on guard cell shape and gas exchange efficiency had been measured previously - as mentioned and cited by the authors (Durney et al. 2023, Liu et al. 2024, Zhang et al. 2022 etc). These experiments confirm that 3xGFP-BdMUTE can dose-dependently rescue stomatal morphology in sid/bdmute and that this correlates with stomatal functionality. Yet, the result that SCs are needed for GC morphology and gas exchange efficiency are not new nor surprising. Therefore, this data does not add much to the main story of the paper and the question how impaired BdMUTE mobility affects cell fate and division orientation to form functional subsidiary cells and guard cells. Likewise, the discussion is rather long but lacks focus on the BdMUTE mobility aspect. My point is that I found the second half of the manuscript rather confusing and would like to ask the authors to streamline it a bit, set data of figure 5 and 6 into clear context to the data in figure 1-4 and have a clear conclusion in their discussion. Right now the discussion ends rather abruptly.

© 2024. Published by The Company of Biologists under the terms of the Creative Commons Attribution License (https://creativecommons.org/licenses/by/4.0/).

Minor points:
The abbreviations MYM, M3GM++, M3GM+, M3GM-, M3GM--, MMY are a lot. I appreciate that Figure 2D has the full construct/plant line's names written out and I used that as reference. However, it is not that obviously displayed. Maybe the authors could add a list of their lines from fully functional BdMUTE (MYM) to weak BdM3GM - - to non-functional (MMY) in Figure 2A including abbreviations? Maybe with some colour code (dark yellow to light yellow or something like that)? I think that could be helpful. Or just assigning numbers e.g. "intensity 1", "intensity 2" etc. could help the reader.

Figure 4A: the sid mutant GCs depicted do not seem to have very skewed ventral walls. There is high variability in stomatal phenotypes and GC division orientation in MMY and sid and most divisions seem to be perfectly "straight" (Figure 4C), however, it may be worth to select a different image that better represents a skewed division for sid (closer to 0.1 than 1.6).

Also related to Figure 4, I find the term "straight" to be not that meaningful, geometrically speaking. Even a "skewed" division can have a straight cell wall. I know what the authors mean, but maybe "longitudinal" or "perpendicular to the GMC walls" helps the reader to grasp what a "normal" symmetric GMC division should look like.

There is a typo in the legend of Figure S1: Shown a(r)e [...]

First revision

Author response to reviewers' comments

Dear Editor and Reviewers

First, we would like to thank all of you for your positive feedback and insightful suggestions. We are convinced that we could address all of them and that the revised manuscript is more streamlined and clearer. Throughout this response, your original comments are in black and our response is in (grass) green.

Reviewer 1 Comments for the Author:
In essence, this manuscript provides an interesting set of data that illuminates the role of BdMUTE during stomatal complexes development. My main criticism concerns the assignment of a GMC-autonomous function during GMC division to BdMUTE. In Fig 2 (see also Results, Section 1), the authors indicate that "mobile signal in the lateral cell files is only visible in the single-tag YFP". Certainly, this signal is seen in SMCs of these lines, but it can also be visualized in SMCs of M3GM+ line (Fig. 2 D). In Fig 3, the signal can be also visualized in SMCs of M3GM++. Because the assigned GMC-autonomous function of BbMUTE rests on the absence of BbMUTE protein in SMCs, the authors should reconsider this point. Could lines M3GM- and/or M3GM-- be used to establish that BdMUTE acts cell-autonomously in guiding GMC division?

We very much appreciate this comment. Indeed, we can reliably visualize the GFP signal in SMCs of M3GM++ lines (Fig. 3) but were not able to do this in M3GM+ lines as suggested by the reviewer. The weak signal visible in SMCs of the M3GM+ line in Fig. 2D is PI rather than GFP signal. To clarify this we added the split channels (FP only, PI only) as panel B in Figure S2 clearly showing that we do not see SMC signal in the M3GM+ lines.
However, the reviewer is correct that even if the signal cannot be visualized, we cannot exclude low, undetectable levels of BdMUTE even in the M3GM-- and M3GM- lines, which could be sufficient to influence division orientation non-cell autonomously (i.e. from the SMC towards the GMC) albeit non-sufficient to induce SC divisions. To address this, we added sentences to the results (lines 444-445) and the discussion (lines 628-631), respectively.

Minor points:
1)PP3, lines 51 and 52: "Grass SC are …" should be supported by a reference.
Done.

© 2024. Published by The Company of Biologists under the terms of the Creative Commons Attribution License (https://creativecommons.org/licenses/by/4.0/).

2)PP3 and 4, lines 68-74: The role of the Arabidopsis bHLH should be succinctly described.
As requested, we added more details regarding the Arabidopsis bHLH on lines 65-67.

3)Fig. 2: The term "aborted" should be defined ("GMC dividing oblique and/or aborted").
Done.

4)PP13, line 375, "has a GC-autonomous role during GC division…" should be replaced by "has an autonomous role during GMC division…"
Done, thanks for spotting this.

5)PP15, line 460, "GC" should be replaced by "GMC".
Done.

6)PP16, line 494, "SC divisions" should be replaced by "SMC divisions".
Done.

7)Fig 3D: "Divided GMC?" should be replaced by "Divided GMC".
Done.

8)PP16, line 498: "guard cell division orientation" should be replaced by "guard mother cell division orientation".
Done.

9)PP16, line 508, "GC and SC divisions" should be replaced by "GMC and SC divisions".
Done.

Reviewer 2 Comments for the Author:
I fully support the hypothesis and the approach authors have taken in this study and would like to emphasise the tremendous amount of well-executed statistical analyses presented here, which is quite impressive - although it sometimes even distracts the reader from the main story. The manuscript is very lengthy and that made it partially confusing for me. Also some of the assumptions (e.g. that the BdMUTE protein is mobile) are not sufficiently explained and backed up by evidence in my opinion.

We would like to thank Reviewer 2 for the positive feedback and their enthusiasm towards the presented work. We recognise that the manuscript is rather lengthy and, therefore, shortened the physiology part to not distract from the main points. However, we decided against removing it altogether as we are very much impressed by the quantitative effect, which nicely cements how (quantitatively!) important grass stomatal form for grass stomatal function is.
We also added one additional data point regarding BdMUTE protein mobility to strengthen the notion that it is indeed the protein itself that is mobile (see below).

Here are some detailed suggestions and requests:

Major points:
1) This manuscript is based on the assumption that the BdMUTE protein is both mobile and functional as long as fusion proteins are only fused to its N-terminus. Hence, a bigger N-terminal fusion protein, such as 3xGFP, should just impair mobility but not function. Unfortunately, 3xGFP did not render BdMUTE immobile and only delayed its migration to and activity in SMCs. Could the authors please elaborate why they assume that the protein and not the BdMUTE mRNA is mobile? If the mRNA is transported into SMCs and translated there, it could explain why this approach with 3xGFP tag did not work as expected. There could be something particular about the 3' end of the BdMUTE mRNA that allows migration to other cells or affects its stability while being transported. Altered mRNA stability in M3GM++ during migration could also account for the lower ratio between nuclear SC and cytoplasmic GMC intensity (Figure 3B). If the authors think that a detailed analysis of how BdMUTE mobility works is beyond the scope here, I can understand, but some evidence should be presented or discussed (if already published elsewhere) that the protein is the mobile

© 2024. Published by The Company of Biologists under the terms of the Creative Commons Attribution License (https://creativecommons.org/licenses/by/4.0/).

part.

Indeed, data on whether the actual protein or the mRNA of mobile transcription factors moves is scarce for most if not all mobile transcription factors described in plants. Usually, it is based on comparing transcriptional to translational reporters, which merely reflects promoter activity and cannot distinguish if protein or mRNA moves. To strengthen our hypothesis that the protein rather than the mRNA moves, we performed whole-mount RNA fluorescence *in-situ* hybridization against the endogenous *BdMUTE* mRNA and could not detect it in SMCs. We added an additional paragraph/sentence in the results (line 454-457) and a detailed protocol in the method section, and added panels A, B and C to Figure S3 to support the notion that the protein rather than the mRNA molecule moves. Nonetheless, we cannot fully exclude that mRNA levels in SMCs are below the detection level, which we also indicated in the manuscript.
Furthermore, our mental picture of mRNAs being small messenger molecules that code for much bigger proteins is wrong (see Milo, R., & Phillips, R. (2015). *Cell Biology by the Numbers,* in particular thispart https://book.bionumbers.org/which-is-bigger-mrna-or-the-protein-it-codes-for/#:~:text=The%20%E2%80%9Cthrea d%2Dlike%E2%80%9D%20mRNA,300%20nm%20(BNID%20100023) and references therein). A single nucleotide is ~330 Da, whereas a single amino acid is on average three times smaller with ~110 Da. In addition, the mRNA contains untranslated regions and a single-amino acid codon consists of three nucleotides. The mRNA is a much bigger molecule and like the protein can form 3D structures. Therefore, the assumption that the smaller protein rather than the much bigger mRNA moves is a fair one even lacking additional data. In fact, estimates suggest that the spatial extent of a given mRNA molecule is 10-fold bigger than the spatial extent of the protein it codes for. We do agree though that this is a very important point to transparently discuss, which is why we added an additional paragraph in the discussion (line 632-639).

2) Since 3xGFP-BdMUTE showed dose-dependent effects, I am missing a control of YFP-BdMUTE (MYM) at lower concentration levels. MYM seems to be a quite strong line and intensity-wise just like M3GM++ (Figure 2 and Figure 3A). Even the addition of one weak MYM line would be enough to show that the 3xGFP tag delays mobility. Without that control, I do not think that impaired mobility can be assumed for M3GM++.

We fully agree with the reviewer that a weak single YFP-BdMUTE line would be a fantastic control. Unfortunately, when YFP-BdMUTE was originally transformed into *sid/bdmute-1* in the Bergmann lab, several lines were recovered but none of them showed any quantitative dosage difference. There was either a strong signal (as for the one used in this study) or no signal at all. This is also part of the reason why we started to carefully quantify the M3xGM lines as the dosage differences allowed us to ask some of the questions we were not able to test before.

3) Overall, the authors present a quite impressive number of statistical analyses (in 6 main figures and 10 supplementary figures) that all seem well executed and conclusive but do not necessarily address the core question about local vs. mobile BdMUTE function they set out to answer. Figure 5 and 6 almost feel like a different story. The effect of subsidiary cells on guard cell shape and gas exchange efficiency had been measured previously - as mentioned and cited by the authors (Durney et al. 2023, Liu et al. 2024, Zhang et al. 2022 etc). These experiments confirm that 3xGFP-BdMUTE can dose-dependently rescue stomatal morphology in sid/bdmute and that this correlates with stomatal functionality. Yet, the result that SCs are needed for GC morphology and gas exchange efficiency are not new nor surprising. Therefore, this data does not add much to the main story of the paper and the question how impaired BdMUTE mobility affects cell fate and division orientation to form functional subsidiary cells and guard cells. Likewise, the discussion is rather long but lacks focus on the BdMUTE mobility aspect. My point is that I found the second half of the manuscript rather confusing and would like to ask the authors to streamline it a bit, set data of figure 5 and 6 into clear context to the data in figure 1-4 and have a clear conclusion in their discussion. Right now the discussion ends rather abruptly.

Thanks for this important comment. While it is true that cited references show failed dumbbell morphology qualitatively these defects were never quantified. In particular, the paired “dumbbell shape” quantification of GCs in stomatal complexes, where only one GC successfully recruited SC is novel and strongly indicates that SC recruitment indeed non-cell-autonomously drives dumbbell morphogenesis. Therefore, what we show in Fig 5 is not a mere dose-dependent rescue of GC morphology but rather that BdMUTE levels in GMCs do not determine GC morphology but the actual

© 2024. Published by The Company of Biologists under the terms of the Creative Commons Attribution License (https://creativecommons.org/licenses/by/4.0/).

recruitment of SCs does. It is true that this was observed and described before in SMC polarity mutants that supposedly express MUTE normally (i.e. Liu et al. 2024 and Zhang et al. 2022). However, none of these studies quantified dumbbell morphology specifically nor tested for MUTE expression. Here, we can clearly show that SCs are required for dumbbell morphogenesis and that this is not controlled by a GMC-autonomous role of MUTE. Nonetheless, we changed the phrasing in our results to clarify and emphasize this (lines 551-555).
Regarding the physiology part, we shortened and streamlined both results and discussion. For example, we removed Fig S7 and S10 and excluded the information and figure panels about iWUE as this is indeed less crucial for the story. We decided against removing the physiology part completely for the following reasons. Here, we managed to quantitatively change morphology without affecting stomatal density as is the case for *sid/bdmute-1* in Raissig et al. 2017 or affecting stomatal cell physiology as is the case in double mutants involving *zmpan2*, which has a role in stomatal kinetics beyond SC formation according to Liu et al. 2024. Thus, here we can quantitatively rescue morphology that quantitatively rescues function.
In addition and as mentioned under point 1, we extended the discussion on BdMUTE mobility (see above).

Minor points:
The abbreviations MYM, M3GM++, M3GM+, M3GM-, M3GM--, MMY are a lot. I appreciate that Figure 2D has the full construct/plant line's names written out and I used that as reference. However, it is not that obviously displayed. Maybe the authors could add a list of their lines from fully functional BdMUTE (MYM) to weak BdM3GM - - to non-functional (MMY) in Figure 2A including abbreviations? Maybe with some colour code (dark yellow to light yellow or something like that)? I think that could be helpful. Or just assigning numbers, e.g. “intensity 1”, “intensity 2” etc. could help the reader.

Thank you for this suggestion. We agree that the abbreviations are hard to follow and, as suggested, added a table to Figure 2 (Fig. 2F).

Figure 4A: the sid mutant GCs depicted do not seem to have very skewed ventral walls. There is high variability in stomatal phenotypes and GC division orientation in MMY and sid and most divisions seem to be perfectly “straight” (Figure 4C), however, it may be worth to select a different image that better represents a skewed division for sid (closer to 0.1 than 1.6).

Thank you for pointing this out. We selected another, more representative image.

Also related to Figure 4, I find the term “straight” to be not that meaningful, geometrically speaking. Even a “skewed” division can have a straight cell wall. I know what the authors mean, but maybe “longitudinal” or “perpendicular to the GMC walls” helps the reader to grasp what a “normal” symmetric GMC division should look like.

Thanks for this input, indeed “straight” is geometrically meaningless when we talk about orientation. As suggested, we used “longitudinal” instead and added a sentence to explain what we mean by it in this context.

There is a typo in the legend of Figure S1: Shown a(r)e [...] Fixed, thanks for spotting it!

<u>Second decision letter</u>

MS ID#: DEVELOP/2024/203011

MS TITLE: Dual role of BdMUTE during stomatal development in the model grass *Brachypodium distachyon*

AUTHORS: Roxane P Spiegelhalder, Lea S Berg, Tiago DG Nunes, Melanie Dörr, Barbara Jesenofsky, Heike Lindner, and Michael T Raissig
ARTICLE TYPE: Research Article

© 2024. Published by The Company of Biologists under the terms of the Creative Commons Attribution License (https://creativecommons.org/licenses/by/4.0/).

I am happy to tell you that your manuscript has been accepted for publication in Development, pending our standard publication integrity checks.

© 2024. Published by The Company of Biologists under the terms of the Creative Commons Attribution License (https://creativecommons.org/licenses/by/4.0/).
